# Investigating the Mediating Role of Cardiometabolic Traits in the Causal Link Between SHBG Levels and Stroke Risk via Network Mendelian Randomization

**DOI:** 10.3390/cimb47070494

**Published:** 2025-06-27

**Authors:** Peijiang Pan, Hao Liang, Mingli Li

**Affiliations:** 1 Guangxi Key Laboratory of AIDS Prevention and Treatment, School of Public Health, Guangxi Medical University, Nanning 530021, China; panpeijiang@stu.gxmu.edu.cn; 2 Biosafety III Laboratory, Life Science Institute, Guangxi Medical University, Nanning 530021, China; 3 Center for Genomic and Personalized Medicine, Guangxi Key Laboratory for Genomic and Personalized Medicine, Guangxi Collaborative Innovation Center for Genomic and Personalized Medicine, Guangxi Medical University, Nanning 530021, China

**Keywords:** sex hormone-binding globulin, stroke, cardiometabolic traits, Mendelian randomization, mediation effect

## Abstract

The causal nature of sex hormone-binding globulin (SHBG) in the pathogenesis of stroke remains uncertain. We explored whether SHBG levels are causally associated with stroke via cardiometabolic traits. A network two-sample Mendelian randomization (MR) study was conducted to determine the mediating roles of cardiometabolic traits in the causal effects of SHBG levels on stroke subtypes. Further two-sample MR analyses were performed to explore the inverse associations between significant cardiometabolic mediators and SHBG levels. The MR results indicated a protective effect of genetically increased SHBG levels on any stroke (odd ratio [OR] = 0.941; 95% confidence interval [CI]: 0.898, 0.984), any ischemic stroke (OR = 0.951; 95% CI: 0.922, 0.981), and small-vessel stroke (OR = 0.871; 95% CI: 0.765, 0.977). Moreover, genetically elevated SHBG levels were associated with lower waist circumference (WC, β = −0.091; 95% CI: −0.136, −0.046), waist-to-hip ratio (WHR, β = −0.057; 95% CI: −0.084, −0.030), triglycerides (TG, β = −0.188; 95% CI: −0.249, −0.127), systolic blood pressure (β = −0.799; 95% CI: −1.068, −0.530), and diastolic blood pressure (β = −0.436; 95% CI: −0.605, −0.267), and a reduced risk of type 2 diabetes mellitus (OR = 0.684; 95% CI: 0.400, 0.968) in both the discovery and replication datasets. The proportions of such cardiometabolic traits that mediated the causal effects of SHBG levels on any stroke, any ischemic stroke, or small-vessel stroke ranged from 17.8% to 52.7%; while the mediating effects of SHBG levels on the causal associations between WC, WHR, and TG and stroke ranged from 18.4% to 68.3%. Our findings suggest a protective effect of genetically elevated SHBG levels on stroke risk via key cardiometabolic mediators, primarily WC, WHR, and TG. The mediating roles of SHBG levels in the causal links from WC, WHR and TG to stroke risk were also established. These pathways support SHBG as a potential biomarker and therapeutic target in stroke prevention.

## 1. Introduction

Globally, nervous system disorders were collectively ranked as the leading cause of disability adjusted life-years (DALYs) in 2021 (441 million), among which the disorder with the highest age-standardized DALYs was stroke, imposing a formidable economic toll and disease burden [1]. Previous studies have demonstrated certain factors, such as doxorubicin, natriuretic peptides, procalcitonin, metabolic disorders, xanthohumol, and blood urea nitrogen-to-albumin ratio, are associated with stroke prevention or prognosis [2,3,4,5]. Possible mechanisms might be related to the calcitonin gene-related peptide inhibiting the senescence-associated secretory phenotype [6], inflammatory pathways [7,8,9], immune responses and metabolic reprogramming [10,11,12], leading to neurotoxicity and stroke. Recently, hormone replacement therapies with testosterone, estrogen plus progestin, or estradiol have been proposed as novel promising tactics to lower the risk of cardiovascular diseases, however, the associations between sex hormones and stroke risk supported by the epidemiological studies of exogenous hormones are controversial, including situations where risks cannot be effectively reduced or increased [13,14,15,16]. This presents the necessity to decipher the causal relationship between sex hormones and their related proteins with stroke.

Sex hormone-binding globulin (SHBG) has traditionally been regarded as a specific binding protein that regulates the bioavailability of circulating free sex hormones, such as 5-alpha-dihydrotestosterone, testosterone, and 17-beta-estradiol [17]. Emerging genome-wide association studies (GWASs) and Mendelian randomization (MR) analyses have provided new insights into the causal associations between genetically determined SHBG levels and type 2 diabetes mellitus (T2DM) [18], coronary heart disease [19], breast cancer [20], asthma [21], and arthritis [22]. Moreover, clinical trials of drugs that increase serum SHBG levels to prevent and treat T2DM and polycystic ovary syndrome have been conducted. The effects of SHBG levels on vascular diseases have been suggested, but only a few studies. A prospective study of 13,192 women in the United States who had gone through menopause showed a significant inverse association between serum SHBG levels and the incidence of ischemic stroke, even after adjusting for T2DM, estradiol and testosterone levels [23]. Nevertheless, conventional epidemiological studies are susceptible to residual confounding, reverse causality, or exposure measurement errors, making the causal associations between SHBG levels and stroke risk elusive or conflicting. Given the limitations of observational studies, MR offers an alternative approach to assess the causality between SHBG levels and stroke outcomes [24,25,26].

Owing to the random assortment of genetic variants at conception, an MR study is conceptually analogous to a randomized controlled trial and overcomes the confounding or reverse causality generated by conventional epidemiological studies [26]. Twin and family studies have indicated that circulating SHBG levels are much more heritable than testosterone, luteinizing hormone, and follicle-stimulating hormone levels, with the heritability estimates ranging from 0.69 to 0.80 [27,28]. More importantly, recent GWASs have identified multiple polymorphisms related to circulating SHBG levels, thus providing a valuable opportunity to explore the causal relationship between SHBG levels and stroke risk using MR approaches. In this current study, we performed a network two-sample MR study with the aim of determining whether SHBG levels are causally associated with stroke and its etiological subtypes via cardiometabolic traits, including obesity, blood pressure, glycemic and lipid phenotypes.

## 2. Materials and Methods

**Figure 1:**
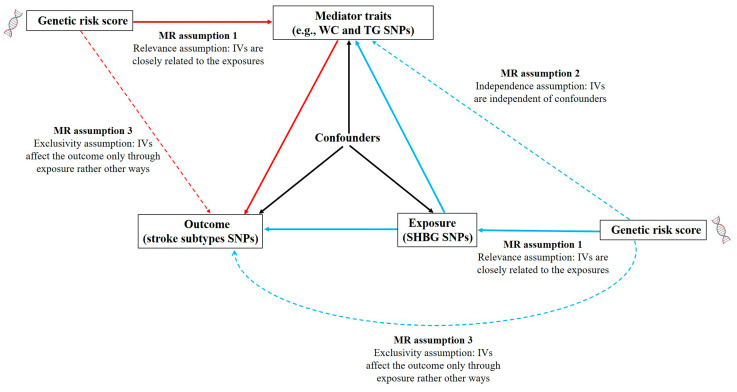
The framework of the network Mendelian randomization (MR) analysis. In this study, we constructed the genetic risk score (GRS) of the significant SNPs related to the exposure as instrument variables (IVs), and performed three two-sample MR tests to estimate the causal associations of exposure–outcome, exposure–mediator, and mediator–outcome. MR analysis should adhere to three core assumptions as follows. Assumption 1: the IVs are strongly associated with the exposure of interest (e.g., SHBG); assumption 2: the IVs should be independent of any confounders correlated with the exposure or outcome; assumption 3: the IVs affect the outcome only through the exposure, rather than any alternative pathways. SHBG, sex hormone-binding globulin; WC, waist circumstance; TG, triglyceride; SNP, single-nucleotide polymorphisms.

All datasets used in this study were obtained from publicly available GWAS summary statistics of populations with European ancestries. Therefore, no ethical approval or written informed consent of the participants was required. Detailed information about the GWAS datasets is provided in Supplementary Table S1. This MR study adhered to the Strengthening the Reporting of Observational Studies in Epidemiology using MR guidelines [29]; the study flowchart and the checklist information are provided in Figure 1 and Supplementary Table S2.

### 2.1. Data Sources

The GWAS summary statistics for SHBG levels were obtained from 312,215 UK Biobank participants of European ancestry. The MEGASTROKE consortium database includes 40,585 cases of any stroke and 406,111 controls of European ancestry; among them, 34,217 had any ischemic stroke, 7193 had cardioembolic stroke, 4373 had large-artery stroke, and 5386 had small-vessel stroke [30]. GWAS summary statistics for intracerebral hemorrhage (1545 cases/1481 controls) were extracted from the International Stroke Genetics Consortium database [31]. For the discovery summary statistics of the cardiometabolic traits, body mass index (BMI), waist circumference (WC), and waist-to-hip ratio (WHR) were retrieved from the Genetic Investigation of Anthropometric Traits (GIANT) consortium [32,33]; the fasting glucose (FG), fasting insulin (FI), glycohemoglobin (HbA1c), and T2DM were retrieved from the Meta-Analyses of Glucose and Insulin-related traits Consortium (MAGIC) and DIAbetes Genetics Replication and Meta-analysis (DIAGRAM) Consortia [34,35,36,37]; the total cholesterol, triglycerides (TG), low-density lipoprotein cholesterol, and high-density lipoprotein cholesterol (HDL-C) were retrieved from the Global Lipids Genetics Consortium (GLGC) [38]; the systolic blood pressure (SBP) and diastolic blood pressure (DBP) were retrieved from the International Consortium for Blood Pressure (ICBP) [39]; and the datasets of hypertension and adiponectin were retrieved from the UK Biobank and the Adiponectin Genetic (ADIPOGen) consortium [40], respectively. For the replication summary statistics of the potential cardiometabolic mediators, the datasets of BMI, FG, and FI were obtained from the European Network for Genetic and Genomic Epidemiology (ENGAGE) Consortium [41]; WC, SBP, and DBP were obtained from the UK Biobank; HbA1c, HDL-C, and TG were acquired from the datasets reported by Prins BP [42]; and WHR and adiponectin were acquired from the datasets reported by Loh PR [43] and Suhre K [44], respectively. Approximately 10% of the glycemic trait samples from the ENGAGE Consortium overlapped with the samples from the MAGIC Consortium, while other GWAS summary statistics were obtained from non-overlapping datasets.

### 2.2. Selection of Instrument Variables (IVs)

We first extracted single-nucleotide polymorphisms (SNPs) associated with exposure at genome-wide significance (*p* < 5 × 10^−8^) from the GWAS summary statistics. These SNPs were further clumped to retain the independent loci using a threshold of linkage disequilibrium (LD) r^2^ > 0.001 based on the 1000 Genomes European reference population panel. Proxy SNPs with high LD (r^2^ > 0.80) were used when SNPs for exposure were not available in the outcome GWAS summary statistics. Next, we harmonized the exposure–outcome datasets with the “Twosample” MR package to allow forward-strand ambiguous SNPs to be inferred from allele frequency information. Furthermore, we computed the variance explained (*R*^2^) and F-statistic for each SNP to quantify the strength of the selected SNPs of an exposure (see Supplementary Methods); F-values greater than 10 are typically recommended for MR analyses [45]. Finally, we combined the selected SNPs associated with the exposure and the weighted genetic risk score (GRS) as IV (which can quantify their increased genetic predisposition) to examine causal inferences for outcomes.

### 2.3. MR Analysis

To identify whether cardiometabolic traits mediate the association between SHBG levels and stroke risk, a network MR analysis, consisting of three two-sample MR tests, was performed to estimate the causal associations of exposure–outcome, exposure–mediator, and mediator–outcome. In step 1, we constructed an SHBG-associated GRS as the IV to estimate the causal effects of genetically determined SHBG levels on any stroke, any ischemic stroke, cardioembolic stroke, large-artery stroke, small-vessel stroke, and intracerebral hemorrhage. In step 2, we explored the causal associations of SHBG levels with cardiometabolic traits using the earliest and largest GWAS summary statistics, and the cardiometabolic traits with a *p*-value of <0.05 were further replicated in the other GWAS summary datasets. In step 3, only the significant cardiometabolic traits that passed a *p*-value of <0.05 in both the discovery and replication datasets were recommended as the potential mediators to infer their causal effects on stroke subtypes. Specific cardiometabolic mediators of the pathways from SHBG levels to stroke risk were identified if their mutual causal associations were supported in all three steps. The mediation ratio was used to estimate the extent to which the total effect contributed to the mediator, and the formula is shown in the Supplementary Methods. Finally, additional MR analyses were performed to determine whether the significant cardiometabolic mediators (WC, WHR, T2DM, TG, SBP, and DBP) were causally associated with SHBG levels in a post hoc analysis.

### 2.4. Statistical Analysis

We examined the MR estimates using the GRS function implemented in the grs.summary module of the R package Genetics ToolboX (version 2.15.1 for Windows; see the Supplementary Methods), which has been previously described [40]. An imbalance in the horizontal pleiotropy of the IV would distort the effect estimate of the exposure and outcome, as well as violate the assumption that the IV of exposure affecting the outcome should be independent of potential confounders. Therefore, multiple sensitivity analyses, including Cochran’s Q test of inverse variance-weighted (IVW, together with the *I*^2^ index and the calculated formula, see the Supplementary Methods), the MR-Egger intercept test, the MR Pleiotropy Residual Sum and Outlier method (MR-PRESSO), and a “leave-one-out” analysis, were performed to assess the robustness and credibility of the MR results. No significant heterogeneity was observed, with a Cochran Q-derived value of *p* > 0.05 and a *I*^2^ < 25% detected by the IVW method. There was no evidence of directional horizontal pleiotropy, with *p* > 0.05 via the MR-Egger intercept test [46]. MR-PRESSO and “leave-one-out” analyses were used to detect potential outlier SNPs. If necessary, we further searched potential pleiotropic effects of each SNP instrument using the GWAS catalog (https://www.ebi.ac.uk/gwas/, accessed on 1 May 2024) and removed pleiotropic SNPs until the pleiotropic test was insignificant. The multiplicative random effects of the IVW (IVW-mre) method, which is often used to address heterogeneity, was used as a complementary analysis to further confirm the causal relationship if the heterogeneity persisted after conducting the aforementioned sensitivity analyses.

For binary outcomes (such as stroke, T2DM, and hypertension), the effect estimates were reported as odds ratios (ORs) with 95% confidence intervals (CIs); for the quantitative outcomes (such as BMI and SBP), the effect estimates were reported as β-values with 95% CIs. All MR analyses were performed using R version 4.3.1 (R Foundation for Statistical Conputing, Vienna, Austria. www.R-project.org/, accessed on 1 May 2024), with two-tailed *p*-values of <0.05 denoting statistical significance.

## 3. Results

### 3.1. A Protective Effect of Genetically Elevated SHBG Levels on Stroke Risk

Of the 264 SNPs associated with SHBG levels at genome-wide significance (*p* < 5 × 10^−8^), 241 SNPs not in LD were included to build the GRS for SHBG, collectively accounting for 12.0% of the total variance. The F-statistic values of the selected SNPs ranged from 30 to 8220, and the F-statistic value of the GRS of SHBG was 177, indicating that the causal estimates are unlikely to be biased by weak IVs (Supplementary Table S3).

**Figure 2:**
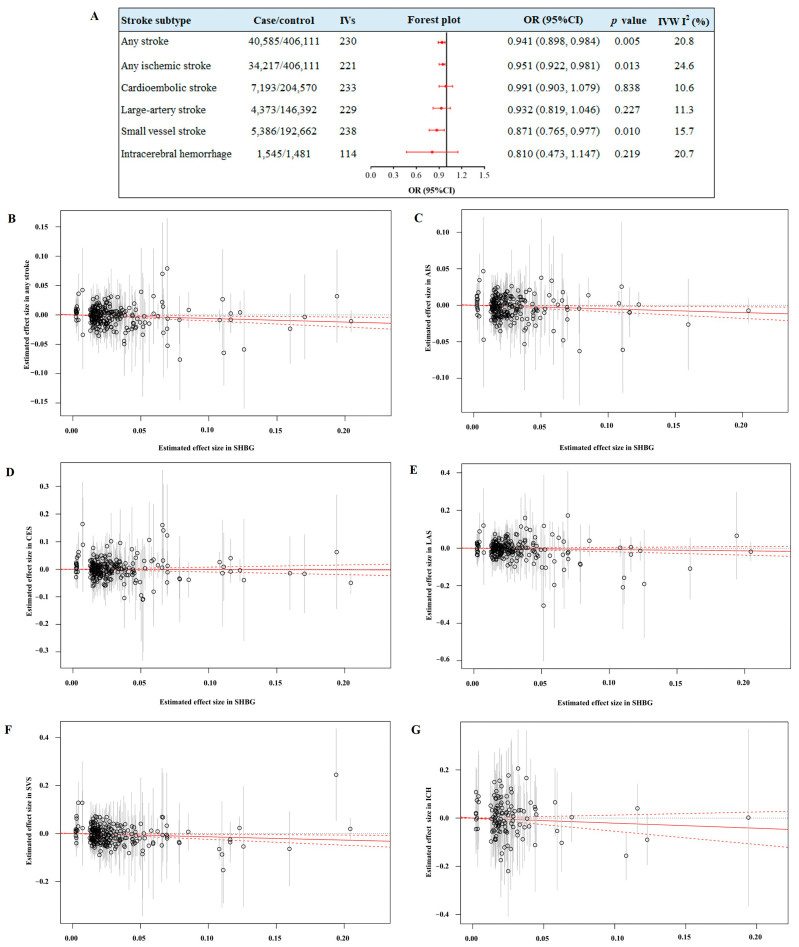
Mendelian randomization associations of sex hormone-binding globulin (SHBG) levels with stroke and related subtypes. (**A**) The forest plots of the causal estimates of SHBG levels with stroke risk by the inverse variance-weighted (IVW) method. The scatter plots of the genetic risk score of SHBG on any stroke (**B**), any ischemic stroke (**C**), cardioembolic stroke (**D**), large-artery stroke (**E**), small-vessel stroke (**F**), and intracerebral hemorrhage (**G**) by the IVW method. The genetic risk scores (GRSs) of the significant SNPs related to SHBG levels were constructed as instrument variables (IVs), and the effect estimates for stroke outcomes were reported as odds ratios (ORs) with 95% confidence intervals (CIs).

As shown in Figure 2, the final IVs of SHBG levels harmonized with stroke subtypes ranged from 114 to 238. Genetically elevated SHBG levels were associated with a lower risk of any stroke (OR = 0.941; 95% CI: 0.898, 0.984; *p =* 0.005), any ischemic stroke (OR = 0.951; 95% CI: 0.922, 0.981; *p =* 0.013), and small-vessel stroke (OR = 0.871; 95% CI: 0.765, 0.977; *p =* 0.010), but not with cardioembolic stroke (OR = 0.991; 95% CI: 0.903, 1.079; *p =* 0.838), large-artery stroke (OR = 0.932; 95% CI: 0.819, 1.046; *p =* 0.227), or intracerebral hemorrhage (OR = 0.810; 95% CI: 0.473, 1.147; *p =* 0.219). After removing the outlier SNPs or the possible pleiotropic SNPs detected by the MR-PRESSO method orGWAS catalog, no significant heterogeneity was observed in these causal associations between SHBG levels and stroke risk (all *I*^2^ < 25% or Cochran Q-derived *p* > 0.05). Furthermore, the MR-Egger intercept method detected no evidence of a directional pleiotropic effect (all *P_intercept_* > 0.05). The results of the MR and sensitivity analyses of SHBG levels and stroke risk are presented in Figure 2 and Supplementary Table S4.

### 3.2. Causal Associations Between SHBG Levels and Cardiometabolic Traits

In the discovery datasets, the final IVs of SHBG levels harmonized with cardiometabolic traits ranged from 48 to 237. MR analyses showed that genetically elevated SHBG levels were associated with lower BMI (β = −0.058; 95% CI: −0.093, −0.023; *p =* 0.001), WC (β = −0.091; 95% CI: −0.136, −0.046; *p =* 5.85 × 10^−5^), WHR (β = −0.057; 95% CI: −0.084, −0.030; *p =* 4.83 × 10^−5^), FG (β = −0.034; 95% CI: −0.054, −0.014; *p =* 0.007), FI (β = −0.028; 95% CI: −0.046, −0.010; *p =* 0.002), TG (β = −0.188; 95% CI: −0.249, −0.127; *p =* 2.22 × 10^−9^), SBP (β = −0.799; 95% CI: −1.068, −0.530; *p =* 5.24 × 10^−9^), DBP (β = −0.436; 95% CI: −0.605, −0.267; *p = 3*.51 × 10^−7^), and reduced risk of T2DM (OR = 0.684; 95% CI: 0.400, 0.968; *p =* 4.77 × 10^−3^), but increased levels of HbA1c (β = 0.020; 95% CI: 0.010, 0.030; *p =* 1.31 × 10^−5^), HDL-C (β = 0.141; 95% CI: 0.094, 0.188; *p =* 5.64 × 10^−9^), and adiponectin (β = 0.037; 95% CI: 0.008, 0.066; *p =* 0.014).

In the replication datasets, the protective effects of SHBG on WC (β = −0.039; 95% CI: −0.057, −0.021; *p =* 2.04 × 10^−5^), WHR (β = −0.065; 95% CI: −0.094, −0.036; *p = 2*.56 × 10^−5^), FG (β = −0.029; 95% CI: −0.049, −0.009; *p =* 0.006), TG (β = −0.131; 95% CI: −0.213, −0.049; *p =* 0.002), SBP (β = −0.055; 95% CI: −0.075, −0.035; *p =* 1.70 × 10^−8^), DBP (β = −0.027; 95% CI: −0.041, −0.013; *p =* 9.37 × 10^−5^), and T2DM (OR = 0.834; 95% CI: 0.749, 0.918; *p =* 2.27 × 10^−5^) were further verified, with the final IVs of SHBG levels ranging from 33 to 189. The causal associations between SHBG levels and cardiometabolic traits are shown in Table 1.

Multiple sensitivity tests were conducted to exclude possible outliers or pleiotropic SNPs, but significant heterogeneities were detected between SHBG and some cardiometabolic traits, including WC, WHR, SBP, DBP, and T2DM. Fortunately, both the IVW-mre method and leave-one-out analysis indicated that these causal estimates were not influenced by any one instrumental variable of SNPs. In addition, the MR-Egger intercept method revealed no directional pleiotropic effect on the associations between SHBG levels and cardiometabolic traits (all *P_intercept_* > 0.05). The results of MR and sensitivity analyses between SHBG levels and cardiometabolic traits are shown in Supplementary Tables S5 and S6 and Supplementary Figures S1–S5.

### 3.3. Causal Pathways from SHBG Levels to Stroke via Cardiometabolic Traits

Based on the findings detected from the discovery and replication GWAS datasets, WC, WHR, FG, T2DM, TG, SBP, and DBP were considered as the potential cardiometabolic mediators of the causal pathways from SHBG levels to stroke risk. We obtained the IVs of the potential cardiometabolic mediators from the discovery GWAS datasets to assess their causal effects on stroke, with the final number of IVs ranging from 10 to 425. All of the IVs were sufficiently strong with an F-statistic value greater than 10. The total explained variance ranged from 1.0% to 9.2% (Supplementary Tables S7–S9).

**Figure 3:**
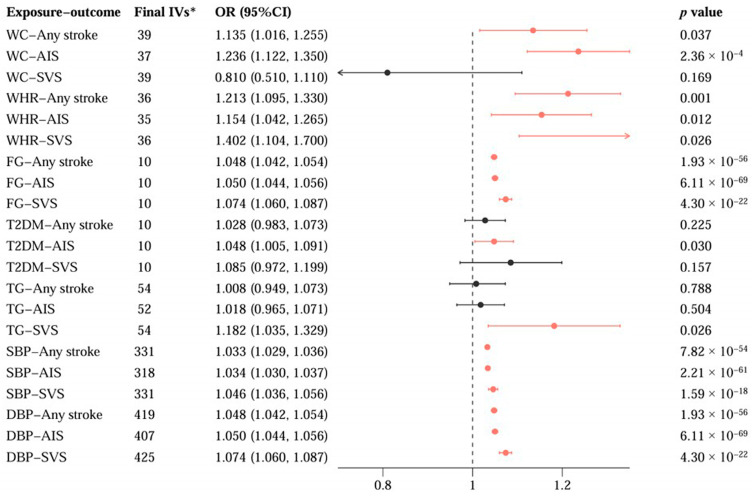
Significant causal associations of the potential cardiometabolic mediators with stroke risk. Based on the significant MR results of sex hormone-binding globulin (SHBG) levels and cardiometabolic mediators in both the discovery and replication GWAS datasets (*p* < 0.05), we identified waist circumference (WC), waist-to-hip ratio (WHR), fasting glucose (FG), type 2 diabetes mellitus (T2DM), triglyceride (TG), systolic blood pressure (SBP), and diastolic blood pressure (DBP) as potential cardiometabolic mediators for the causal pathways from SHBG to the risk of any stroke, any ischemic stroke (AIS), and small-vessel stroke (SVS). Herein, we selected the instrumental variables (IVs) of the potential cardiometabolic mediators from the discovery GWAS datasets to assess their causal effects on stroke risk. * After removal of any potential pleiotropic and outlier SNPs for the exposure, along with the SNPs that were not available in the outcome summary statistics, the final SNPs were included to build a genetic risk score for MR analyses. OR, odds ratio; CI, confidence interval; GWAS, genome wide association study.

**Figure 4:**
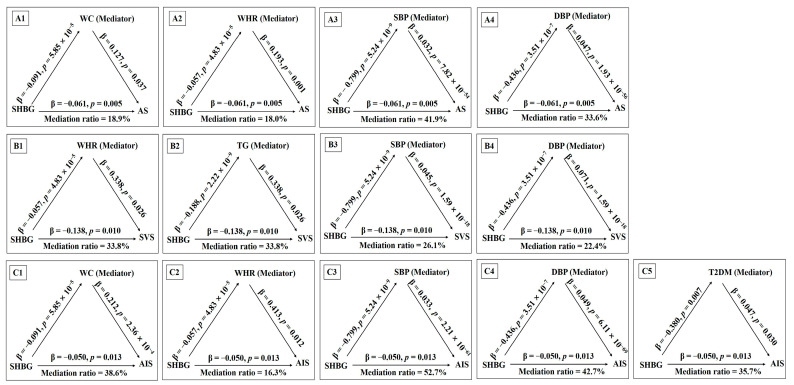
The mediating effects of cardiometabolic traits on the causal links from sex hormone-binding globulin (SHBG) to stroke risk. The effects of SHBG on any stroke (AS) mediated by WC (**A1**), WHR (**A2**), SBP (**A3**), and DBP (**A4**). The effects of SHBG on small-vessel stroke (SVS) mediated by WHR (**B1**), TG (**B2**), SBP (**B3**), and DBP (**B4**). The effects of SHBG on any ischemic stroke (AIS) mediated by WC (**C1**), WHR (**C2**), SBP (**C3**), DBP (**C4**), and T2DM (**C5**). WC, waist circumference; WHR, waist-to-hip ratio; SBP, systolic blood pressure; DBP, diastolic blood pressure; TG, triglyceride; T2DM, type 2 diabetes mellitus.

The MR network analyses suggested that genetically determined WHR, SBP, and DBP may be the common mediators of the associations between SHBG levels with any stroke, ischemic stroke, and small-vessel stroke, and the mediating effects ranged from 17.8% to 52.7%. Furthermore, genetically determined higher WC had a positive impact on any stroke (OR = 1.135; 95%CI: 1.016, 1.255; *p =* 0.037) and ischemic stroke (OR = 1.236; 95%CI: 1.122, 1.350; *p =* 2.36 × 10^−4^), contributing 18.9% and 38.6% to the total effect of SHBG levels on any stroke and ischemic stroke, respectively. Genetic susceptibility to T2DM and TG were positively associated with any ischemic stroke (OR = 1.048; 95%CI: 1.005, 1.091; *p =* 0.030) and small-vessel stroke (OR = 1.182; 95%CI: 1.035, 1.329; *p =* 0.026), explaining 35.7 and 22.8% of the total effect of SHBG levels on any ischemic stroke and small-vessel stroke, respectively. The significant causal diagrams and mediating results are presented in Figure 3 and Figure 4.

The MR-Egger intercept method showed no significant directional pleiotropic effects between the potential cardiometabolic mediators and stroke (all *P_intercept_* > 0.05). Although significant heterogeneities were observed in the associations between WC with any stroke and small-vessel stroke, WHR with any stroke and ischemic stroke, SBP and DBP with any stroke and ischemic stroke, both the IVW-mre and “leave-one-out” analyses indicated that their causal estimates were not be altered by any one instrumental variable of SNPs (Supplementary Tables S7–S9 and Supplementary Figures S6–S9).

### 3.4. Causal Effects of the Cardiometabolic Mediators on Stroke Risk via SHBG

**Figure 5:**
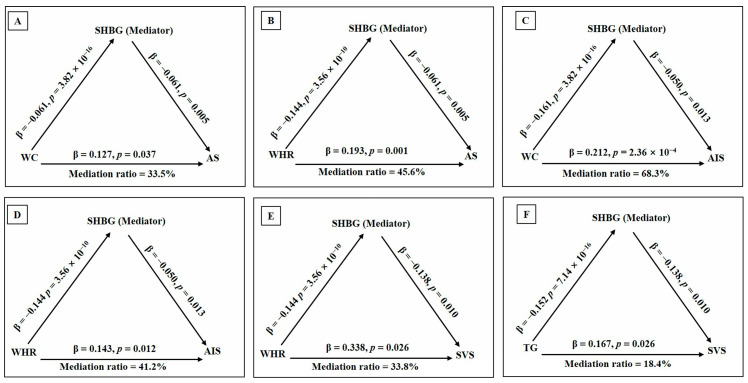
The mediating effects of SHBG on the causal links from cardiometabolic traits to stroke risk. The effects of WC (**A**) and WHR (**B**) on any stroke (AS) mediated by SHBG. The effects of WC (**C**) and WHR (**D**) on any ischemic stroke (AIS) mediated by SHBG. The effects of WHR (**E**) and TG (**F**) on small-vessel stroke (SVS) mediated by SHBG. WC, waist circumference; WHR, waist-to-hip ratio; TG, triglyceride; SHBG, sex hormone-binding globulin.

To further examine whether SHBG also mediated the causal effects of cardiometabolic traits on stroke, we assessed the causal associations between the potential cardiometabolic mediators and SHBG levels using IVs in the discovery datasets (for the MR design, see Supplementary Figure S10). The final IVs of the cardiometabolic mediators harmonized with SHBG ranged from 12 to 368, and genetically elevated levels of WC (β = −0.161; 95% CI: −0.200, −0.122; *p =* 3.82 × 10^−16^), WHR (β = −0.144; 95% CI: −0.189, −0.099; *p =* 3.56 × 10^−10^), TG (β = −0.152; 95% CI: −0.189, −0.115; *p =* 7.14 × 10^−16^), SBP (β = −0.003; 95% CI: −0.005, −0.002; *p =* 1.03 × 10^−5^), and T2DM (β = −0.071; 95% CI: −0.091, −0.051; *p =* 4.27 × 10^−13^) resulted in lower SHBG levels. Furthermore, genetically determined SHBG levels mediated the greater effects on the associations between WC and WHR with any stroke, any ischemic stroke, and small-vessel stroke, and the mediating ratios ranged from 33.5 to 68.3%. In addition, SHBG accounted for 18.4% of the total effect of TG on small-vessel stroke, whereas the mediating effects of SHBG on the associations between T2DM and SBP with stroke risk were too low to be noteworthy (Figure 5 and Supplementary Table S10).

There was no directional pleiotropic effect observed in the associations between cardiometabolic traits and SHBG using the MR-Egger intercept method (all *P_intercept_* > 0.05). Although significant heterogeneities were detected in the relationship between some cardiometabolic traits with SHBG levels, the IVW-mre and “leave-one-out” analyses indicated that the causal estimates were not influenced by any one instrumental variable of SNPs. This suggests that SHBG also plays a key role in mediating the causal associations between WC, WHR, and TG with stroke risk. The results of the sensitivity analyses are presented in Supplementary Table S10 and Supplementary Figures S11–S15.

## 4. Discussion

To our best knowledge, this is the first study using a network MR framework to investigate the causal pathways from SHBG levels to stroke risk via cardiometabolic traits. Based on large-scale GWAS summary statistics, we found that genetically elevated SHBG levels were associated with a lower risk of any stroke, any ischemic stroke, and small-vessel stroke, along with lower WC, WHR, TG, SBP and DBP and a reduced risk of T2DM in both the discovery and replication datasets. Furthermore, these aforementioned cardiometabolic traits also mediated the causal links between SHBG levels and the risk of any stroke, any ischemic stroke, or small-vessel stroke, with the mediating effects ranging from 17.8 to 52.7%. In the reverse MR direction, the mediating proportions of SHBG levels in the causal associations between WC, WHR, and TG with any stroke, any ischemic stroke, or small-vessel stroke ranged from 18.4 to 68.3%. Multiple sensitivity analyses further confirmed the robustness and the credibility of these findings.

Consistent with the results of a prospective cohort study in postmenopausal women [23], a protective effect of genetically determined SHBG levels on ischemic stroke was detected in the present study, particularly for the etiologic subtype of small-vessel stroke. Our findings were also supported by previous data showing that increased circulating SHBG levels were linked to lower risks of metabolic syndrome and cardiovascular events [47,48,49], thus reducing the risk of stroke. In addition, SHBG could markedly suppress lipopolysaccharide-induced inflammatory biomarkers in macrophages and adipocytes, such as monocyte chemoattractant protein-1, TNFα, and IL-6 [50], which have been reported to be closely related to stroke risk and prognosis [51,52]. Although the sample size of the GWASs was relatively large, our study did not detect a significant relationship between genetically determined SHBG levels with cardioembolic or large-artery strokes. Meanwhile, we did not identify a significant association between genetically determined SHBG levels and intracerebral hemorrhage, which is attributed to small-vessel disease; the possible reason may be a lack of statistical power because of the small sample size of cases of intracerebral hemorrhage (*n* = 3026). Further studies with larger samples are thus essential to explore whether circulating levels of SHBG are more important in small-vessel disease than other etiological subtypes.

Related studies have shown that metabolic disorders, including obesity, blood pressure, dyslipidemia, and glycemic traits, are closely associated with circulating SHBG levels and stroke risk [53,54,55]. Therefore, we hypothesized that these conventional cardiometabolic traits may be involved in the causal pathway from SHBG level to stroke risk. As expected, our study found genetically determined SHBG levels were causally associated with a lower risk of WC, WHR, T2DM, TG, SBP, and DBP, which further reduced the risk of any stroke, ischemic stroke, or small-vessel stroke. The underlying mechanisms between SHBG and metabolic disorders or stroke may involve activation of the inflammatory response [56,57], accelerating lipid accumulation in macrophages and adipocytes [50], or increasing free testosterone with downstream pro-androgenic effects [58]. Contrary to some studies [47,59] showing that serum SHBG levels were inversely related to the individual components of metabolic syndrome, except for blood pressure, our results supported the notion that genetic predisposition to higher SHBG levels were favorable with SBP and DBP, and these two traits mediated the greater effects of genetically determined SHBG on any stroke or ischemic stroke. Moreover, the causal effects of the genetic determinants of SBP, DBP, and antihypertensive drugs on the risk of various stroke etiologies have been well established [60]. Collectively, we considered that WC, WHR, T2DM, TG, SBP, and DBP could partially mediate the pathways from lower SHBG levels to the risk of any stroke, ischemic stroke, or small-vessel stroke.

In the reverse MR direction, our results demonstrated an inverse causal association between WC, WHR, and TG levels with SHBG levels. This suggests elevated WC, WHR, and TG in turn lower circulating SHBG levels, increasing the stroke risk. Previous studies have linked excessive fat accumulation, especially abdominal obesity represented by WC and WHR, with hepatotoxicity and cardiac and pulmonary dysfunction via excessive activation of oxidative stress and immunological/inflammatory effects [61,62,63], and then leads to synthesis and secretion disorders of SHBG in the liver. In addition, monosaccharide-induced lipogenesis could downregulate HNF-4α levels in HepG2 cells, which in turn reduced hepatic SHBG expression [64]. Our findings and the aforementioned biological explanations collectively strengthen the evidence supporting SHBG as a promising therapeutic target for stroke and metabolic disorders. Some physical non-drug interventions, such as walking, swimming, jogging, cycling, weight-training, smoking cessation, and salt reduction, have been recommended to effectively improve these causal associations.

Our study has some methodological strengths. The comprehensive and large-scale GWAS summary datasets enhance the statistical power and accuracy of the associations, especially for binary traits (such as stroke). In addition, the MR results were validated using multiple sensitivity analyses for possible pleiotropy and outlier SNPs, overcoming the limitations of conventional epidemiological studies with respect to reverse causality and confounding to some degree. Moreover, performing two-sample MR analyses with different samples could lower the risk of the effect-size estimate of the SNP with the strongest association, which tends to be overestimated in a one-sample GWAS, a phenomenon known as the winner’s curse [65].

Nevertheless, our study had several limitations. First, a well-conducted and unbiased MR study should comply with three main assumptions. Fortunately, the high F-values of the SNP-GRSs suggesting each IV has a strong association with the exposures of interest, and multiple sensitivity approaches indicated no directional pleiotropic effects observed in exposures or outcomes. Although marginal heterogeneities were observed in some cardiometabolic traits (e.g., WC, WHR, SBP, and DBP) with SHBG or stroke, both of the IVW-mre and “leave-one-out” analyses found that the causal estimates were not influenced by any one instrumental variable of SNP. Second, as the measured SHBG levels and stroke diagnostic criteria may be heterogeneous across ancestries, our study only included European individuals to minimize population stratification, which would limit generalizability to some degree. Further studies involving extensive populations are thus essential. Third, we merely used summary-level data in our MR analyses because of the limited publicly available individual-level data; therefore, the possibility of a non-linear causal relationship or interaction association between SHBG levels and risk of stroke or cardiometabolic traits cannot be ruled out. Lastly, we could not conduct sex-stratified analysis of SHBG levels and stroke due to data unavailability.

## 5. Conclusions

Our study highlighted a protective effect of genetically increased SHBG levels on stroke risk via key cardiometabolic mediators, primarily WC, WHR, and TG; moreover, the mediating roles of SHBG levels in the causal links from WC, WHR and TG to stroke risk were also established. These causal findings suggest SHBG as a promising biomarker and therapeutic target in stroke prevention.

## Figures and Tables

**Table 1:** Causal associations of sex hormone-binding globulin levels with cardiometabolic traits in the discovery and replication datasets.

Outcomes	Causal Estimates in the Discovery Datasets			Causal Estimates in the Replication Datasets ^#^		
	Sample Sizes	OR/β (95% CI) *	IVW *p* Value	Sample Sizes	OR/β (95% CI) *	IVW *p* Value
BMI	322,154	−0.058 (−0.093, −0.023)	0.001	87,048	−0.005 (−0.046, 0.036)	0.820
WC	232,101	−0.091 (−0.136, −0.046)	5.85 × 10^−5^	336,639	−0.039 (−0.057, −0.021)	2.04 × 10^−5^
WHR	210,082	−0.057 (−0.084, −0.030)	4.83 × 10^−5^	502,773	−0.065 (−0.094, −0.036)	2.56 × 10^−5^
FG	41,486	−0.034 (−0.054, −0.014)	0.007	87,048	−0.029 (−0.049, −0.009)	0.006
FI	51,750	−0.028 (−0.046, −0.010)	0.002	87,048	−0.017 (−0.039, 0.005)	0.123
HbA1c	46,368	0.020 (0.010, 0.030)	1.31 × 10^−5^	9436	−0.040 (−0.095, 0.015)	0.162
T2DM	69,033	0.684 (0.400, 0.968)	4.77 × 10^−3^	298,957	0.834 (0.749, 0.918)	2.27 × 10^−5^
TC	94,595	−0.023 (−0.080, 0.034)	0.221	NA	NA	NA
TG	94,595	−0.188 (−0.249, −0.127)	2.22 × 10^−9^	9796	−0.131 (−0.213, −0.049)	0.002
LDL-C	94,595	−0.027 (−0.068, 0.014)	0.201	NA	NA	NA
HDL-C	94,595	0.141 (0.094, 0.188)	5.64 × 10^−9^	403,943	0.103 (−0.024, 0.230)	0.113
SBP	757,601	−0.799 (−1.068, −0.530)	5.24 × 10^−9^	436,419	−0.055 (−0.075, −0.035)	1.70 × 10^−8^
DBP	757,601	−0.436 (−0.605, −0.267)	3.51 × 10^−7^	436,424	−0.027 (−0.041, −0.013)	9.37 × 10^−5^
Hypertension	361,194	1.000 (0.999, 1.000)	0.857	NA	NA	NA
Adiponectin	39,883	0.037 (0.008, 0.066)	0.014	1000	0.207 (−0.020, 0.434)	0.075

* For binary outcomes (e.g., T2DM, hypertension), the effect estimate was reported as odds ratio (OR) with 95% confidence interval (CI). For quantitative outcomes (e.g., BMI, SBP, DBP, and TG), the effect estimate was reported as β value with 95% CI. ^#^ Only the significant cardiometabolic traits with a *p*-value of <0.05 in the discovery datasets were further analyzed in the replication datasets. IVW, inverse variance-weighted; BMI, body mass index; WC, waist circumference; WHR, waist and hip ratio; FG, fasting glucose; FS, fasting insulin; HbA1c, glycosylated hemoglobin; T2DM, type 2 diabetes mellitus; TC, total cholesterol; TG, triglyceride; LDL-C, low-density lipoprotein cholesterol; HDL-C, high-density lipoprotein cholesterol; SBP, systolic blood pressure; DBP, diastolic blood pressure; NA, not available.

## Data Availability

The datasets used and analyzed in this study are publicly available and provided at the locations cited in the main text.

## Supplementary Materials

The following supporting information can be downloaded at: https://www.mdpi.com/article/10.3390/cimb47070494/s1. References [66,67,68,69,70,71] are cited in the Supplementary Materials.
