# Supplementary material for: Investigating the Mediating Role of Cardiometabolic Traits in the Causal Link Between SHBG Levels and Stroke Risk via Network Mendelian Randomization
Source: Curr Issues Mol Biol. 2025 Jun 27;47(7):494. doi: 10.3390/cimb47070494 (PMC12293703; doi:10.3390/cimb47070494)
Supplement: Supplementary file 1 [file cimb-47-00494-s001.zip › cimb-3662914-supplementary.pdf]

Supplementary Materials

Investigating the Mediating Role of Cardiometabolic Traits in the Causal Link Between SHBG Levels and Stroke Risk via Network Mendelian Randomization

Title	Page (s)
Supplementary methods	3-6
Supplementary Table S1. Genome wide association study (GWAS) summary statistics used in this study	7-9
Supplementary Table S2. STROBE-MR checklist of recommended items to address in reports of Mendelian randomization studies	10-13
Supplementary Table S3. Genome-wide significant ($P < 1\times10^{-8}$) SNPs associated with sex hormone-binding globulin levels	14-20
Supplementary Table S4. Mendelian randomization and sensitivity analyses between sex hormone-binding globulin levels and stroke risk	21
Supplementary Table S5. Mendelian randomization and sensitivity analyses between sex hormone-binding globulin levels and cardiometabolic traits in the discovery datasets	22
Supplementary Table S6. Mendelian randomization and sensitivity analyses between sex hormone-binding globulin levels and cardiometabolic traits in the replication datasets	23
Supplementary Table S7. Mendelian randomization and sensitivity analyses between the potential cardiometabolic mediators and any stroke	24
Supplementary Table S8. Mendelian randomization and sensitivity analyses between the potential cardiometabolic mediators and any ischemic stroke	25
Supplementary Table S9. Mendelian randomization and sensitivity analyses between the potential cardiometabolic mediators and small vessel stroke	26
Supplementary Table S10. Mendelian randomization and sensitivity analyses between the cardiometabolic mediators and sex hormone-binding globulin levels	27
Supplementary Figure S1. Leave-one-out analysis for the causal estimates of sex hormone-binding globulin and waist circumference in the discovery dataset (A) and replication dataset (B)	28
Supplementary Figure S2. Leave-one-out analysis for the causal estimates of sex hormone-binding globulin and waist-to-hip ratio in the discovery dataset (A) and replication dataset (B)	29
Supplementary Figure S3. Leave-one-out analysis for the causal estimates of sex hormone-binding globulin and systolic blood pressure in the discovery dataset (A) and replication dataset (B)	30
Supplementary Figure S4. Leave-one-out analysis for the causal estimates of sex hormone-binding globulin and diastolic blood pressure in the discovery dataset (A) and replication dataset (B)	31
Supplementary Figure S5. Leave-one-out analysis for the causal estimates of sex hormone-binding globulin and type 2 diabetes mellitus in the discovery dataset (A) and replication dataset (B)	32
Supplementary Figure S6. Leave-one-out analysis for the causal estimates of waist circumference with any	33

stroke (A) and small-vessel stroke (B)	
Supplementary Figure S7. Leave-one-out analysis for the causal estimates of waist-to-hip ratio with any stroke (A) and any ischemic stroke (B)	34
Supplementary Figure S8. Leave-one-out analysis for the causal estimates of systolic blood pressure with any stroke (A) and any ischemic stroke (B)	35
Supplementary Figure S9. Leave-one-out analysis for the causal estimates of diastolic blood pressure with any stroke (A) and any ischemic stroke (B)	36
Supplementary Figure S10. Study design of the causal effects of the cardiometabolic mediators on stroke risk via sex hormone-binding globulin	37
Supplementary Figure S11. Leave-one-out analysis for the causal estimates of waist circumference and sex hormone-binding globulin	38
Supplementary Figure S12. Leave-one-out analysis for the causal estimates of waist-to-hip ratio and sex hormone-binding globulin	39
Supplementary Figure S13. Leave-one-out analysis for the causal estimates of triglyceride and sex hormone-binding globulin	40
Supplementary Figure S14. Leave-one-out analysis for the causal estimates of systolic blood pressure and sex hormone-binding globulin	41
Supplementary Figure S15. Leave-one-out analysis for the causal estimates of diastolic blood pressure and sex hormone-binding globulin	42
Supplementary references	43

Supplementary methods

The variance explained for the exposure of interest

The variance explained (conceptually similar to R^2) for the exposure of interest was calculated for each single nucleotide polymorphism (SNP) according to the formula as follows [1].

$$R^2 = \frac{2\beta^2 \times MAF \times (1 - MAF)}{2\beta^2 \times MAF \times (1 - MAF) + \left(se(\beta)\right)^2 \times 2N \times MAF \times (1 - MAF)}$$

Where MAF is the minimum allele frequency, β is the effect estimate of the SNP, and se (β) is the standard error of the effect estimate. Total variance explained was calculated in an additive model assuming no interaction between the individual SNPs.

The F statistic for single SNP

The F-statistic for single instrument SNP was calculated using the formula as follows [2].

$$F = \frac{\beta^2}{se_\beta^2}$$

Where β is the effect estimate of the SNP, and se (β) is the standard error of the effect estimate.

The *F* statistic for the SNP-genetic risk score (GRS)

The F-statistic for the SNP-GRS was calculated using the formula as follows [3].

$$F = \frac{R^2 \times (N - m - 1)}{(1 - R^2) \times m}$$

Where R^2 is the proportion of the variance of the phenotype that is explained by the instruments, N represents the sample size of the phenotype, m is the number of instruments of the phenotype.

The mediation analysis

The extent to which the association of SHBG with stroke mediated by the mediators was tested in a post hoc analysis after the specific metabolic traits were identified as the potential mediators, and the calculated formula is detailed illustrated in previous study [4]. For example, the total effect (odds ratio: OR) of 1-SD increase of SHBG on any stroke was 0.94 [ln (OR) = -0.061]. The effect of 1-SD increase of SHBG on WC was -0.091, and 1 unit increase in WC was associated with any stroke was 1.14 [ln (OR) = 0.127]. Therefore, the mediated effect of WC was -0.091 × 0.127 = -0.012. The mediation ratio was -0.012/-0.061*100 = 18.9%.

The *grs.summary* module

We examined the MR estimates using the GRS function implemented in th*e grs.summary* module of the R package *Genetics ToolboX* (version 2.15.1 for Windows). The grs.summary module approximates the regression of an outcome onto an additive GRS, using only single SNP association summary statistics extracted from GWAS results. For uncorrelated SNPs, when maximizing the likelihood function, the αIV value and its standard error (se_{α}) can be estimated with the formula as follows [5].

$$\alpha \cong \frac{\sum \omega \times \beta \times se_{\beta}^{-2}}{\sum \omega^2 \times se_{\beta}^{-2}}$$

$$se_{\alpha} \cong \sqrt{\frac{1}{\sum \omega^2 \times se_{\beta}^{-2}}}$$

Where ω denotes the estimated effects on the exposure, β values are estimated effects on the outcome, with standard errors se_{β}.

Cochran's Q I^2 index

The I^2 index [6, 7], defined as the percentage of total variance in the estimates to be pooled explained by heterogeneity rather than sampling error, is related to the Cochran's Q statistic through the following formula:

$$I^2 = \begin{cases} \frac{Q-(m-1)}{Q} \times 100, & for\ Q \geq m-1 \\ 0, & for\ Q < m-1 \end{cases}$$

Where m is corresponding to the number of instrument SNPs, Q value was directly obtained from Cochran's Q test of inverse variance-weighted. When $I^2 < 25\%$ indicates no significant heterogeneity, $I^2 <$ ranges from 25% to 50% indicates moderate heterogeneity, and $I^2 > 50\%$ indicates severe heterogeneity.

The R programming script used in this study

Example for SHBG and stroke, other analyses of cardiometabolic traits and SHBG or stroke risk could be conducted in the same way

```
### Get list of all available studies
   library(googleAuthR)
   library(remotes)
   library(devtools)
   library(ieugwasr)
   library(TwoSampleMR)
### association between SHBG and stroke risk
  ## extract exposure_SHBG
   exposure_dat <- extract_instruments("ukb-d-30830_irnt")
   exposure_dat <- clump_data(exposure_dat, clump_r2 = 0.001)
  ## extract outcome_stroke
   # any stroke
   outcome_dat <- extract_outcome_data(snps=exposure_dat$SNP, outcomes="ebi-a-GCST006906")
   # any ischemic stroke
   outcome_dat <- extract_outcome_data(snps=exposure_dat$SNP, outcomes="ebi-a-GCST005843")
   # cardioembolic stroke
   outcome_dat <- extract_outcome_data(snps=exposure_dat$SNP, outcomes="ebi-a-GCST006910")
   # large artery stroke
   outcome_dat <- extract_outcome_data(snps=exposure_dat$SNP, outcomes="ebi-a-GCST006907")
   # small-vessel stroke
   outcome_dat <- extract_outcome_data(snps=exposure_dat$SNP, outcomes="ebi-a-GCST006909")
   # Intracerebral hemorrhage
   outcome_dat <- extract_outcome_data(snps=exposure_dat$SNP, outcomes="ebi-a-GCST005843")
   outcome_txt <- read.table("./ICH_all.txt",head=TRUE)
   outcome_dat<-format_data(outcome_txt, type="outcome",
                    snps = exposure_dat$p_dat,
                    snp_col = "MarkerName",
                    beta_col = "Effect",
                    se_col = "StdErr",
                    effect_allele_col = "Allele1",
                    other_allele_col = "Allele2",
                    pval_col = "P-value"
     )
###  harmonise data
   dat <- harmonise_data(exposure_dat, outcome_dat)
###Heterogeneity statistics
   mr_heterogeneity(dat)
### Horizontal pleiotropy
   mr_pleiotropy_test(dat)
###MR-PRESSO
```

```
   if (!require("devtools")) { install.packages("devtools") } else {}
   devtools::install_github("rondolab/MR-PRESSO")
   library(MRPRESSO)
    mr_presso(BetaOutcome = "beta.outcome", BetaExposure = "beta.exposure",SdOutcome =
     "se.outcome",
          SdExposure = "se.exposure",OUTLIERtest = TRUE,DISTORTIONtest = TRUE,
          data = dat, NbDistribution = 1000,  SignifThreshold = 0.05)
### GRS
   library(gtx)
   grs <- grs.summary(dat[,"beta.exposure"], dat[,"beta.outcome"], dat[,"se.outcome"], n)
   # n is the sample of the outcome

   View(grs)
    grs.plot(dat[,"beta.exposure"], dat[,"beta.outcome"], dat[,"se.outcome"],text = NULL, textpos =
    NULL, textcex = 0.5,
         alpha = 0.05)
   title(xlab = "Effect size in ln(SHBG)", ylab = "Effect size in any stroke (lnOR)")
### scatter plot
   res <- mr(dat, method_list=c("mr_egger_regression", "mr_ivw","mr_weighted_median"))
   generate_odds_ratios(res)
   p <- mr_scatter_plot(res, dat)
   p[[1]]
###funnel plot
   res_single <- mr_singlesnp(dat)
   p <- mr_funnel_plot(res_single)
   p[[1]]
```

Supplementary Table S1 Genome wide association study (GWAS) summary statistics used in this study

	Discovery GWAS summary statistics						Replication GWAS summary statistics					
**Phenotype**	**Data source**	**Sample size or case/control**	**Males (%)**	**Smoker (%)**	**Taking medicine (%)**	**URL for data**	**Data source**	**Sample size or case/ control**	**Male (%)**	**Smoker (%)**	**Taking medicine (%)**	**URL for data**
AS	MEGASTROKE Consortium	40,585/406,111	53.9	23.3%	NA	http://megastroke.org/download.html3	/	/	/	/	/	/
AIS	MEGASTROKE Consortium	34,217/406,111	58.3	NA	NA	http://megastroke.org/download.html3	/	/	/	/	/	/
CES	MEGASTROKE Consortium	7,193/406,111	53.6	NA	NA	http://megastroke.org/download.html3	/	/	/	/	/	/
LAS	MEGASTROKE Consortium	4,373/406,111	51.1	NA	NA	http://megastroke.org/download.html3	/	/	/	/	/	/
SVS	MEGASTROKE Consortium	5,386/192,662	54.5	NA	NA	http://megastroke.org/download.html3	/	/	/	/	/	/
ICH	Woo, 2014	1,545/1,481	45.0	NA	NA	http://cerebrovascularportal.org/informational/downloads4	/	/	/	/	/	/
SHBG	UK Biobank	312,215	NA	NA	NA	https://gwas.mrcieu.ac.uk/datasets/ukb-d-30830_irnt/	/	/	/	/	/	/
BMI	GIANT Consortium	322,154	47.5	NA	NA	http://portals.broadinstitute.org/collaboration/giant/index.php/GIANT_consortium_data_files	ENGAGE Consortium	87,048	42.8	NA	NA	http://diagram-onsortium.org/2015_ENGAGE_1KG/
WC	GIANT Consortium	232,101	44.5	NA	NA	http://portals.broadinstitute.org/collaboration/giant/index.php/GIANT_consortium_data_files	UK Biobank	336,639	NA	NA	NA	https://gwas.mrcieu.ac.uk/datasets/ukb-a-382/

WHR	GIANT Consortium	210,082	44.5	NA	NA	http://portals.broadinstitute.org/collaboration/giant/index.php/GIANT_consortium_data_files	Loh PR, 2018	502,773	NA	NA	NA	https://gwas.mrcieu.ac.uk/datasets/ebi-a-GCST90029009/
FG	MAGIC	41,486	47.5	NA	NA	https://magicinvestigators.org/downloads/	ENGAGE Consortium	87,048	42.8	NA	NA	http://diagram-consortium.org/2015_ENGAGE_1KG/
FI	MAGIC	51,750	47.4	NA	NA	https://magicinvestigators.org/downloads/	ENGAGE Consortium	87,048	42.8	NA	NA	http://diagram-consortium.org/2015_ENGAGE_1KG/
HbA1c	MAGIC	46,368	48.0	NA	NA	https://magicinvestigators.org/downloads/	Prins BP, 2017	9,436	NA	NA	NA	https://gwas.mrcieu.ac.uk/datasets/ebi-a-GCST004939/
T2DM	DIAGRAM Consortium	12,171/56,862	49.9	NA	NA	https://gwas.mrcieu.ac.uk/datasets/ebi-a-GCST006867/	Mahajan A, 2018	48,286/ 250,671	NA	NA	NA	https://gwas.mrcieu.ac.uk/datasets/ebi-a-GCST007515/
TC	GLGC	94,595	52.0	NA	NA	http://csg.sph.umich.edu/willer/public/lipids2013/	/	/	/	/	/	/
TG	GLGC	94,595	52.0	NA	NA	http://csg.sph.umich.edu/willer/public/lipids2013/	Prins BP, 2017	9,796	NA	NA	NA	https://gwas.mrcieu.ac.uk/datasets/ebi-a-GCST005073/

LDL-C	GLGC	94,595	52.0	NA	NA	http://csg.sph.umich.edu/willer/public/lipids2013/	/	/	/	/	/	/
HDL-C	GLGC	94,595	52.0	NA	NA	http://csg.sph.umich.edu/willer/public/lipids2013/	UK Biobank	403,943	NA	NA	NA	https://gwas.mrcieu.ac.uk/datasets/ieu-b-109/
SBP	ICBP Consortium	757,601	45.8	NA	NA	https://gwas.mrcieu.ac.uk/datasets/ieu-b-38/	UK Biobank	436,419	NA	NA	NA	https://gwas.mrcieu.ac.uk/datasets/ukb-b-20175/
DBP	ICBP Consortium	757,601	45.8	NA	NA	https://gwas.mrcieu.ac.uk/datasets/ieu-b-39/	UK Biobank	436,424	NA	NA	NA	https://gwas.mrcieu.ac.uk/datasets/ukb-b-7992/
Hypertension	UK Biobank	1,237/35,9957	NA	NA	NA	https://gwas.mrcieu.ac.uk/datasets/ukb-d-I9_HYPTENS/	/	/	/	/	/	/
Adiponectin	ADIPOGen Consortium	39,883	NA	NA	NA	https://www.mcgill.ca/genepi/adipogen-consortium	Suhre K, 2017	1,000	NA	NA	NA	https://gwas.mrcieu.ac.uk/datasets/prot-c-3554_24_1/

Data source: GIANT, the Genetic Investigation of Anthropometric Traits; MAGIC, the Meta-Analyses of Glucose and Insulin-related traits Consortium; DIAGRAM, DIAbetes Genetics Replication and Meta-analysis; GLGC, Global Lipids Genetics Consortium; ICBP, the International Consortium for Blood Pressure; ADIPOGen, Adiponectin Genetic; ENGAGE, the European Network for Genetic and Genomic Epidemiology.

Abbreviation: NA, not available; AS, Any stroke; AIS, Any ischemic stroke; CES, cardioembolic stroke; LAS, large-artery stroke; SVS, small vessel stroke; ICH, intracerebral hemorrhage; SHBG, sex hormone-binding globulin; BMI, body mass index; WC, waist circumference; WHR, waist-to-hip ratio; FG, fasting glucose; FS, fasting insulin; HbA1c, glycosylated hemoglobin; T2DM, type 2 diabetes mellitus; TC, total cholesterol; TG, triglyceride; LDL-C, low-density lipoprotein cholesterol; HDL-C, high-density lipoprotein cholesterol; SBP, systolic blood pressure; DBP, diastolic blood pressure.

Supplementary Table S2 STROBE-MR checklist of recommended items to address in reports of Mendelian randomization studies[1,2]

Item No.	Section		Checklist item	Complete/location
1	**TITLE and ABSTRACT**		Indicate Mendelian randomization (MR) as the study's design in the title and/or the abstract if that is a main purpose of the study	Title and abstract
	**INTRODUCTION**			
2	**Background**		Explain the scientific background and rationale for the reported study. What is the exposure? Is a potential causal relationship between exposure and outcome plausible? Justify why MR is a helpful method to address the study question	Introduction, Paragraphs 1-3
3	**Objectives**		State specific objectives clearly, including pre-specified causal hypotheses (if any). State that MR is a method that, under specific assumptions, intends to estimate causal effects	Introduction, Paragraph 3
	**METHODS**			
4	**Study design and data sources**		Present key elements of the study design early in the article. Consider including a table listing sources of data for all phases of the study. For each data source contributing to the analysis, describe the following:	
		a)	Setting: Describe the study design and the underlying population, if possible. Describe the setting, locations, and relevant dates, including periods of recruitment, exposure, follow-up, and data collection, when available.	Materials and methods, paragraphs 1-2 (sections: Data sources)
		b)	Participants: Give the eligibility criteria, and the sources and methods of selection of participants. Report the sample size, and whether any power or sample size calculations were carried out prior to the main analysis	Materials and methods, paragraphs 2-3 (sections: Data sources, Selection of IVs).
		c)	Describe measurement, quality control and selection of genetic variants	Materials and methods, paragraphs 2-4 (sections: Data sources, Selection of IVs, MR analysis).
		d)	For each exposure, outcome, and other relevant variables, describe methods of assessment and diagnostic criteria for diseases	Materials and methods, paragraphs 2-4 (sections: Data sources, Selection of IVs, MR analysis). Further information is provided in Supplementary methods and Supplementary Table S1
		e)	Provide details of ethics committee approval and participant informed consent, if relevant	Not relevant
5	**Assumptions**		Explicitly state the three core IV assumptions for the main analysis (relevance, independence and exclusion restriction) as well assumptions for any additional or sensitivity analysis	Materials and methods, paragraphs 3-5 (sections: Selection of IVs, MR analysis, Statistical analysis). Detail information is provided in Figure 1
6	**Statistical methods: main analysis**		Describe statistical methods and statistics used	
		a)	Describe how quantitative variables were handled in the analyses (i.e., scale, units, model)	Materials and methods, paragraph 5 (sections: Statistical analysis).

		b) Describe how genetic variants were handled in the analyses and, if applicable, how their weights were selected	Materials and methods, paragraphs 3-5 (sections: Selection of IVs, MR analysis, Statistical analysis).
		c) Describe the MR estimator (e.g. two-stage least squares, Wald ratio) and related statistics. Detail the included covariates and, in case of two-sample MR, whether the same covariate set was used for adjustment in the two samples	Materials and methods, paragraphs 3-5 (sections: Selection of IVs, MR analysis, Statistical analysis).
		d) Explain how missing data were addressed	Not applicable
		e) If applicable, indicate how multiple testing was addressed	Materials and methods, paragraph 5 (sections: Statistical analysis)
7	**Assessment of assumptions**	Describe any methods or prior knowledge used to assess the assumptions or justify their validity	Materials and methods, paragraphs 3-5 (sections: Selection of IVs, MR analysis, Statistical analysis).
8	**Sensitivity analyses and additional analyses**	Describe any sensitivity analyses or additional analyses performed (e.g. comparison of effect estimates from different approaches, independent replication, bias analytic techniques, validation of instruments, simulations)	Materials and methods, paragraph 5 (sections: Statistical analysis)
9	**Software and pre-registration**		
		a) Name statistical software and package(s), including version and settings used	Materials and methods, paragraph 5 (sections: Statistical analysis)
		b) State whether the study protocol and details were pre-registered (as well as when and where)	Not applicable
	**RESULTS**		
10	**Descriptive data**		
		a) Report the numbers of individuals at each stage of included studies and reasons for exclusion. Consider use of a flow diagram	Materials and methods, paragraph 2 (sections: Data Data sources). Further information is provided in Supplementary Table S1
		b) Report summary statistics for phenotypic exposure(s), outcome(s), and other relevant variables (e.g. means, SDs, proportions)	Materials and methods, paragraph 2 (sections: Data sources). Further information is provided in Supplementary Table S1
		c) If the data sources include meta-analyses of previous studies, provide the assessments of heterogeneity across these studies	Not applicable
		d) For two-sample MR:	
		i. Provide justification of the similarity of the genetic variant-exposure associations between the exposure and outcome samples	Materials and methods, paragraph 2 (sections: Data sources), Supplementary Table S1
		ii. Provide information on the number of individuals who overlap between the exposure and outcome studies	Not applicable
11	**Main results**		
		a) Report the associations between genetic variant and exposure, and between genetic variant and outcome, preferably on an interpretable scale	Results: sections 1~3, Supplementary Table S3, Supplementary Tables 7~9

		b)	Report MR estimates of the relationship between exposure and outcome, and the measures of uncertainty from the MR analysis, on an interpretable scale, such as odds ratio or relative risk per SD difference	Results: sections 1~4, Figures 2~5, Table 1, Supplementary Tables 4~10
		c)	If relevant, consider translating estimates of relative risk into absolute risk for a meaningful time period	Not applicable
		d)	Consider plots to visualize results (e.g. forest plot, scatterplot of associations between genetic variants and outcome versus between genetic variants and exposure)	Figures 2~5, Supplementary Figures 1~9 Supplementary Figures 11~15
12	**Assessment of assumptions**			
		a)	Report the assessment of the validity of the assumptions	Results: sections 1~4, Supplementary Table S3, Supplementary Tables 7~9
		b)	Report any additional statistics (e.g., assessments of heterogeneity across genetic variants, such as I^2, Q statistic or E-value)	Supplementary Tables 4~10
13	**Sensitivity analyses and additional analyses**			
		a)	Report any sensitivity analyses to assess the robustness of the main results to violations of the assumptions	Results: sections 1~4, Supplementary Tables 4~10
		b)	Report results from other sensitivity analyses or additional analyses	Results: sections 1~4, Supplementary Tables 4~10 Supplementary Figures 1~9 Supplementary Figures 11~15
		c)	Report any assessment of direction of causal relationship (e.g., bidirectional MR)	Results: sections 2~4, Supplementary Tables 4~10 Supplementary Figures 1~15
		d)	When relevant, report and compare with estimates from non-MR analyses	Not applicable
		e)	Consider additional plots to visualize results (e.g., leave-one-out analyses)	Figures 2~4 Supplementary Figures 1~9 Supplementary Figures 11~15
	**DISCUSSION**			
14	**Key results**		Summarize key results with reference to study objectives	Discussion, paragraph 1
15	**Limitations**		Discuss limitations of the study, taking into account the validity of the IV assumptions, other sources of potential bias, and imprecision. Discuss both direction and magnitude of any potential bias and any efforts to address them	Discussion, paragraph 6
16	**Interpretation**			
		a)	Meaning: Give a cautious overall interpretation of results in the context of their limitations and in comparison with other studies	Discussion, paragraphs 2~6

		b)	Mechanism: Discuss underlying biological mechanisms that could drive a potential causal relationship between the investigated exposure and the outcome, and whether the gene-environment equivalence assumption is reasonable. Use causal language carefully, clarifying that IV estimates may provide causal effects only under certain assumptions	Discussion, paragraphs 2~6
		c)	Clinical relevance: Discuss whether the results have clinical or public policy relevance, and to what extent they inform effect sizes of possible interventions	Discussion, paragraphs 2~6
17	**Generalizability**		Discuss the generalizability of the study results (a) to other populations, (b) across other exposure periods/timings, and (c) across other levels of exposure	Discussion, paragraphs 2~6
	**OTHER INFORMATION**			
18	**Funding**		Describe sources of funding and the role of funders in the present study and, if applicable, sources of funding for the databases and original study or studies on which the present study is based	Funding
19	**Data and data sharing**		Provide the data used to perform all analyses or report where and how the data can be accessed, and reference these sources in the article. Provide the statistical code needed to reproduce the results in the article, or report whether the code is publicly accessible and if so, where	Data availability statement
20	**Conflicts of Interest**		All authors should declare all potential conflicts of interest	Declaration of competing interest

This checklist is copyrighted by the Equator Network under the Creative Commons Attribution 3.0 Unported (CC BY 3.0) license.

Supplementary Table S3 Genome-wide significant ($P < 1×10^{-8}$) SNPs associated with sex hormone-binding globulin levels

Chr	SNP	Position	Effect allele	Other allele	EAF	Beta	SE	*P* value	Variance explained (R^2)	F statistic
1	rs2196944	214359057	G	C	0.64	-0.0262	0.0024	1.72E-28	0.0004	123
1	rs35504625	154599778	G	C	0.48	0.0242	0.0023	1.20E-26	0.0004	114
1	rs2642438	220970028	G	A	0.70	0.0252	0.0025	2.93E-24	0.0003	103
1	rs3850873	51062976	G	A	0.43	0.0165	0.0023	6.18E-13	0.0002	52
1	rs2281003	171106709	C	T	0.47	-0.0159	0.0023	2.45E-12	0.0002	49
1	rs71514237	23824647	T	G	0.69	0.0150	0.0025	1.88E-09	0.0001	36
1	rs7535528	2444414	A	G	0.37	0.0140	0.0024	2.85E-09	0.0001	35
1	rs6703810	39345358	C	T	0.08	0.0239	0.0043	2.07E-08	0.0001	31
1	rs164895	91569329	A	T	0.71	-0.0138	0.0025	4.11E-08	0.0001	30
1	rs4639796	197126649	A	G	0.16	-0.0272	0.0031	1.36E-18	0.0002	77
1	rs115276619	184865132	A	T	0.02	-0.0593	0.0091	6.79E-11	0.0001	43
1	rs2494196	219762581	A	C	0.29	0.0161	0.0025	1.44E-10	0.0001	41
1	rs1870927	226426337	A	T	0.62	0.0140	0.0024	2.65E-09	0.0001	35
1	rs114165349	27021913	C	G	0.02	-0.1597	0.0075	1.07E-99	0.0014	450
1	rs267733	150958836	G	A	0.16	-0.0248	0.0031	8.29E-16	0.0002	65
1	rs28549287	110230138	A	G	0.77	-0.0236	0.0029	1.22E-15	0.0002	64
1	rs6603985	93017159	T	A	0.79	0.0218	0.0028	5.24E-15	0.0002	61
1	rs2791644	11160674	T	C	0.77	-0.0174	0.0027	9.92E-11	0.0001	42
1	rs1762509	107592959	G	A	0.66	-0.0437	0.0024	2.67E-74	0.0011	333
1	rs1497406	16505320	G	A	0.58	0.0235	0.0023	1.01E-24	0.0003	105
1	rs9970140	61684288	G	A	0.08	0.0378	0.0043	8.76E-19	0.0003	78
1	rs144647926	235467607	A	G	0.09	0.0284	0.0040	1.93E-12	0.0002	50
1	rs182698541	9993359	C	T	0.08	0.0244	0.0042	4.58E-09	0.0001	34
1	rs6675469	26789813	A	G	0.21	-0.0157	0.0028	1.61E-08	0.0001	32
1	rs79094524	40041371	GA	G	0.24	-0.0270	0.0027	5.44E-24	0.0003	102
1	rs35346083	25788425	A	C	0.44	-0.0175	0.0023	1.81E-14	0.0002	59
1	rs10753556	23732762	G	A	0.88	0.0223	0.0034	7.75E-11	0.0001	42
1	rs4847406	93758450	T	C	0.50	-0.0138	0.0023	1.24E-09	0.0001	37
1	rs7536494	205162434	G	C	0.88	-0.0207	0.0035	1.88E-09	0.0001	36
2	rs2138161	227095159	C	T	0.65	-0.0285	0.0024	2.13E-33	0.0005	145
2	rs10191389	191588979	C	G	0.38	-0.0189	0.0024	9.24E-16	0.0002	65
2	rs72948328	111881810	T	C	0.06	-0.0325	0.0048	9.44E-12	0.0001	46
2	rs7557569	65644561	C	T	0.41	0.0148	0.0023	1.40E-10	0.0001	41
2	rs57467915	220081416	A	G	0.02	-0.0514	0.0092	2.64E-08	0.0001	31
2	rs10184004	165508389	T	C	0.41	0.0257	0.0023	8.04E-29	0.0004	124
2	rs1260326	27730940	C	T	0.61	0.0697	0.0023	7.48E-199	0.0029	906
2	rs6736913	42510018	G	A	0.98	-0.0659	0.0079	5.19E-17	0.0002	70
2	rs2160348	64909166	C	T	0.74	-0.0447	0.0026	3.15E-67	0.0010	300
2	rs1375045	112263087	A	G	0.24	0.0157	0.0027	3.59E-09	0.0001	35

2	rs71401561	27363063	CT	C	0.48	-0.0129	0.0023	2.80E-08	0.0001	31
2	rs6758199	70537173	T	C	0.07	-0.0439	0.0046	9.27E-22	0.0003	92
2	rs13029936	208465544	T	C	0.18	0.0228	0.0029	6.86E-15	0.0002	61
2	rs2305144	70441775	C	A	0.41	-0.0161	0.0023	4.12E-12	0.0002	48
2	rs77358763	234613877	C	T	0.07	-0.0274	0.0045	1.72E-09	0.0001	36
2	rs13035806	178091822	A	G	0.12	-0.0200	0.0035	1.70E-08	0.0001	32
3	rs79287178	172294500	A	G	0.03	-0.0657	0.0068	7.15E-22	0.0003	92
3	rs2953782	129739633	C	T	0.77	0.0150	0.0027	3.66E-08	0.0001	30
3	rs6792725	24520283	G	A	0.69	0.0295	0.0025	2.49E-31	0.0004	136
3	rs4518111	12377344	C	A	0.56	0.0246	0.0023	1.30E-26	0.0004	114
3	rs34261514	49745941	AC	A	0.32	-0.0246	0.0024	2.76E-24	0.0003	103
3	rs57158761	185371172	G	A	0.44	-0.0156	0.0023	1.03E-11	0.0001	46
3	rs7615115	105490068	T	G	0.67	0.0152	0.0024	3.11E-10	0.0001	40
3	rs13094241	196190893	G	T	0.73	0.0160	0.0025	3.56E-10	0.0001	39
3	rs687339	135932359	T	C	0.77	-0.0626	0.0027	1.09E-118	0.0017	537
3	rs113986045	135742057	GTGT TTTG TTT	G	0.22	0.0225	0.0027	2.09E-16	0.0002	68
3	rs9844972	150097635	C	G	0.07	-0.0302	0.0045	1.68E-11	0.0001	45
4	rs13150068	88203828	G	A	0.44	-0.0397	0.0023	1.75E-67	0.0010	301
4	rs1126670	100052733	A	C	0.70	-0.0247	0.0025	1.65E-23	0.0003	100
4	rs17547712	171180561	A	G	0.08	0.0286	0.0043	2.01E-11	0.0001	45
4	rs4691375	157650930	G	A	0.32	0.0157	0.0024	1.06E-10	0.0001	42
4	rs999634	52927113	A	C	0.58	0.0129	0.0023	2.01E-08	0.0001	31
4	rs13108218	3443931	G	A	0.62	-0.0452	0.0024	2.56E-82	0.0012	370
4	rs28925904	144359490	T	C	0.03	-0.0517	0.0072	9.79E-13	0.0002	51
4	rs10027275	148981496	C	G	0.74	-0.0248	0.0026	1.99E-21	0.0003	90
4	rs11736110	110017182	T	C	0.05	0.0354	0.0051	2.69E-12	0.0002	49
4	rs17011177	86830058	T	G	0.16	0.0179	0.0031	8.16E-09	0.0001	33
4	rs7696472	69538180	A	G	0.48	0.0263	0.0023	4.94E-31	0.0004	134
4	rs78890745	159834474	A	G	0.11	0.0388	0.0036	1.98E-26	0.0004	113
4	rs28507491	77197651	A	G	0.38	0.0232	0.0023	2.83E-23	0.0003	99
4	rs2011603	18025484	A	G	0.74	-0.0171	0.0026	3.22E-11	0.0001	44
4	rs62294491	969111	T	C	0.11	-0.0206	0.0037	2.51E-08	0.0001	31
4	rs34168560	69325415	G	C	0.32	0.0310	0.0025	2.28E-36	0.0005	159
4	rs4568225	23898430	T	C	0.31	0.0165	0.0024	1.31E-11	0.0001	46
4	rs56257546	3001689	C	G	0.03	0.0415	0.0065	1.94E-10	0.0001	41
5	rs11743810	137802404	T	C	0.56	0.0165	0.0023	5.18E-13	0.0002	52
5	rs79760705	53298716	T	G	0.11	-0.0313	0.0036	2.65E-18	0.0002	76
5	rs6860245	127367998	C	G	0.25	0.0205	0.0026	7.42E-15	0.0002	60
5	rs11948950	55876283	A	G	0.11	-0.0268	0.0038	1.97E-12	0.0002	50
5	rs1981811	72910810	A	G	0.45	0.0134	0.0023	4.45E-09	0.0001	34
5	rs40270	55804552	C	A	0.77	-0.0360	0.0028	9.91E-38	0.0005	165

5	rs10069690	1279790	T	C	0.26	-0.0156	0.0026	1.85E-09	0.0001	36
5	rs258495	75038901	A	T	0.63	-0.0131	0.0024	2.61E-08	0.0001	31
5	rs1651274	158020425	G	A	0.23	-0.0185	0.0027	9.35E-12	0.0001	46
5	rs329122	133864599	A	G	0.42	-0.0136	0.0023	3.47E-09	0.0001	35
6	rs3130487	31723389	T	C	0.85	-0.0301	0.0032	3.48E-21	0.0003	89
6	rs9375703	130386971	G	T	0.67	-0.0181	0.0024	8.09E-14	0.0002	56
6	rs998584	43757896	A	C	0.48	-0.0161	0.0023	1.27E-12	0.0002	50
6	rs6939861	41703041	A	G	0.26	-0.0289	0.0026	1.90E-28	0.0004	122
6	rs1408270	25873184	G	A	0.27	0.0202	0.0026	2.89E-15	0.0002	62
6	rs4709746	164133001	T	C	0.13	0.0198	0.0033	3.38E-09	0.0001	35
6	rs668871	160769811	T	C	0.47	0.0325	0.0023	2.70E-46	0.0007	204
6	rs9379084	7231843	A	G	0.12	-0.0330	0.0037	1.60E-19	0.0003	82
6	rs3813498	108944165	T	C	0.81	-0.0178	0.0029	1.04E-09	0.0001	37
6	rs9349624	52247638	G	A	0.23	0.0152	0.0027	2.02E-08	0.0001	31
6	rs35694679	34243641	ACTCT	A	0.96	-0.0416	0.0060	3.36E-12	0.0002	48
7	rs7015	97920623	G	A	0.81	0.0693	0.0029	3.94E-125	0.0018	567
7	rs10278546	100516003	C	A	0.19	0.0315	0.0029	5.01E-28	0.0004	120
7	rs114949263	150498245	C	T	0.11	0.0319	0.0036	9.46E-19	0.0003	78
7	rs4610628	1903100	T	C	0.37	-0.0187	0.0023	1.72E-15	0.0002	63
7	rs1708302	28198677	T	C	0.50	0.0173	0.0023	2.37E-14	0.0002	58
7	rs58429317	77128987	A	G	0.28	0.0149	0.0025	4.81E-09	0.0001	34
7	rs11556924	129663496	T	C	0.39	0.0174	0.0023	6.24E-14	0.0002	56
7	rs10245356	101906354	T	C	0.62	0.0149	0.0023	1.88E-10	0.0001	41
7	rs3173833	150491084	G	T	0.57	0.0138	0.0023	1.91E-09	0.0001	36
7	rs112100676	86989355	T	A	0.04	0.0348	0.0061	1.48E-08	0.0001	32
7	rs1229492	81564122	C	T	0.73	-0.0195	0.0026	3.71E-14	0.0002	57
7	rs1352084	46265136	C	A	0.70	0.0146	0.0025	3.14E-09	0.0001	35
7	rs157935	130585553	G	T	0.30	0.0205	0.0025	1.03E-16	0.0002	69
7	rs10238028	99208899	G	A	0.07	0.0331	0.0046	3.79E-13	0.0002	53
7	rs878521	44255643	A	G	0.25	-0.0143	0.0026	4.81E-08	0.0001	30
8	rs117921873	81473835	G	A	0.07	0.0531	0.0046	1.10E-30	0.0004	133
8	rs77258375	42332640	CT	C	0.36	0.0211	0.0024	9.03E-19	0.0003	78
8	rs2721195	145677011	C	T	0.53	-0.0194	0.0023	1.27E-17	0.0002	73
8	rs371744786	81340551	CTT	C	0.15	0.0272	0.0033	9.30E-17	0.0002	69
8	rs7828742	116960729	G	A	0.60	-0.0187	0.0023	9.40E-16	0.0002	65
8	rs13254494	23414743	T	C	0.58	-0.0141	0.0023	8.41E-10	0.0001	38
8	rs76120024	12623287	GT	G	0.33	-0.0134	0.0024	3.89E-08	0.0001	30
8	rs9987289	9183358	G	A	0.91	0.0408	0.0039	2.40E-25	0.0003	108
8	rs9297994	59392324	A	G	0.66	0.0238	0.0024	3.39E-23	0.0003	98
8	rs117734486	81061523	T	C	0.06	-0.0345	0.0048	6.25E-13	0.0002	52
8	rs113976091	81870786	G	A	0.05	0.0373	0.0054	6.43E-12	0.0002	47
8	rs6998725	81438093	A	T	0.16	0.0474	0.0031	4.66E-52	0.0007	231

9	rs9697210	131468740	A	G	0.15	-0.0321	0.0032	2.16E-23	0.0003	99
9	rs7860634	139089679	A	G	0.57	0.0197	0.0023	7.70E-18	0.0002	74
9	rs820504	6668278	A	G	0.14	-0.0227	0.0033	6.45E-12	0.0002	47
9	rs62580777	113042911	C	T	0.18	0.0203	0.0030	8.14E-12	0.0001	47
9	rs10868080	86626769	A	T	0.74	-0.0463	0.0026	5.14E-71	0.0010	318
9	rs568656	4133874	C	A	0.35	0.0201	0.0025	3.12E-16	0.0002	67
9	rs34525966	137268044	C	T	0.78	-0.0281	0.0027	1.34E-24	0.0003	105
9	rs7041363	117146043	G	C	0.49	0.0179	0.0023	5.00E-15	0.0002	61
9	rs10984972	123409864	C	T	0.70	-0.0167	0.0025	1.44E-11	0.0001	46
10	rs10822161	65118203	A	G	0.47	0.1080	0.0023	1.00E-200	0.0072	2280
10	rs12263369	94823343	T	C	0.59	-0.0196	0.0023	2.08E-17	0.0002	72
10	rs2792751	113940329	C	T	0.73	-0.0162	0.0025	2.04E-10	0.0001	40
10	rs79717793	5262267	A	G	0.16	-0.0441	0.0031	4.88E-45	0.0006	198
10	rs10881987	93641869	G	A	0.53	0.0196	0.0023	6.31E-18	0.0002	74
10	rs72834770	64603244	A	T	0.02	-0.0474	0.0073	1.12E-10	0.0000	42
10	rs3818708	104503584	T	C	0.47	0.0124	0.0023	4.36E-08	0.0000	30
10	rs34736335	64047444	G	A	0.09	0.0349	0.0040	5.60E-18	0.0000	75
10	rs10882714	97827752	C	T	0.36	0.0145	0.0024	7.78E-10	0.0000	38
10	rs3824655	13370779	C	G	0.59	0.0132	0.0023	9.43E-09	0.0000	33
10	rs1892501	81097266	G	A	0.19	-0.0319	0.0029	7.81E-29	0.0000	124
10	rs4948487	63669865	A	C	0.48	-0.0178	0.0023	4.25E-15	0.0000	62
10	rs2254069	122875589	A	G	0.12	-0.0249	0.0035	9.32E-13	0.0000	51
11	rs12575636	95311260	G	T	0.19	-0.0227	0.0029	5.88E-15	0.0000	61
11	rs9332817	118365210	C	G	0.03	0.0445	0.0071	3.33E-10	0.0000	39
11	rs78730126	48046368	T	C	0.08	0.0256	0.0042	1.02E-09	0.0000	37
11	rs35139828	82740002	A	G	0.18	-0.0169	0.0030	1.32E-08	0.0000	32
11	rs10832570	16249510	G	A	0.39	-0.0209	0.0023	2.95E-19	0.0000	80
11	rs12805041	69284560	T	C	0.23	0.0326	0.0027	7.24E-34	0.0000	147
11	rs3842763	2179204	T	G	0.24	0.0267	0.0027	4.41E-23	0.0000	98
11	rs1675090	61940106	A	G	0.54	0.0128	0.0023	1.99E-08	0.0000	32
11	rs145678014	32927778	T	G	0.05	0.0378	0.0055	5.28E-12	0.0002	48
11	rs1037169	13361005	C	T	0.69	-0.0255	0.0025	2.55E-25	0.0003	108
11	rs174554	61579463	G	A	0.35	-0.0245	0.0024	7.78E-25	0.0003	106
11	rs10750766	65473798	A	C	0.71	-0.0238	0.0025	1.53E-21	0.0003	91
11	rs2155380	119080037	G	A	0.27	0.0180	0.0026	1.93E-12	0.0002	50
11	rs10902121	306791	C	T	0.33	-0.0150	0.0024	5.51E-10	0.0001	38
12	rs7314285	111522026	G	T	0.07	0.0696	0.0045	6.77E-54	0.0008	239
12	rs76895963	4384844	G	T	0.02	0.1258	0.0087	2.29E-47	0.0007	209
12	rs12320328	25408464	G	A	0.08	-0.0466	0.0041	5.03E-30	0.0004	130
12	rs11045806	21314736	A	G	0.19	0.0251	0.0029	7.40E-18	0.0002	74
12	rs11110390	100874901	T	C	0.33	-0.0164	0.0024	8.12E-12	0.0001	47
12	rs12313762	57692470	T	C	0.24	0.0321	0.0026	5.46E-34	0.0005	148
12	rs10745941	102835693	T	C	0.76	-0.0208	0.0027	7.58E-15	0.0002	60

12	rs1716407	124515218	A	G	0.59	-0.0165	0.0023	9.06E-13	0.0002	51
12	rs6488910	124393697	T	G	0.34	0.0138	0.0024	9.95E-09	0.0001	33
12	rs7306128	21132187	G	A	0.05	-0.0282	0.0051	2.48E-08	0.0001	31
12	rs938434	103557947	G	A	0.54	-0.0222	0.0023	4.86E-22	0.0003	93
12	rs61066360	46071336	G	A	0.19	-0.0177	0.0029	1.82E-09	0.0001	36
12	rs73079476	21343833	C	A	0.15	-0.0580	0.0032	8.61E-75	0.0011	335
12	rs2393791	121423956	T	C	0.62	0.0338	0.0023	3.08E-47	0.0007	208
12	rs61932613	51258838	T	G	0.36	0.0187	0.0024	2.79E-15	0.0002	62
12	rs56196860	2908330	A	C	0.03	0.0434	0.0065	2.24E-11	0.0001	45
12	rs10876431	53730160	A	G	0.08	-0.0241	0.0042	1.21E-08	0.0001	32
12	rs10841521	20587192	G	A	0.27	0.0147	0.0026	1.36E-08	0.0001	32
12	rs141881700	101056213	A	G	0.01	-0.1101	0.0104	4.58E-26	0.0004	112
13	rs36179992	50639586	GTA	G	0.51	0.0165	0.0023	1.41E-12	0.0002	50
13	rs9316500	51094114	G	T	0.29	-0.0158	0.0025	2.12E-10	0.0001	40
13	rs35678857	95222890	A	C	0.35	0.0193	0.0024	5.54E-16	0.0002	66
13	rs61968020	114764535	C	G	0.37	-0.0147	0.0025	2.85E-09	0.0001	35
14	rs72683923	50735947	C	T	0.02	0.0666	0.0081	1.45E-16	0.0002	68
14	rs12879423	25927832	G	A	0.68	-0.0164	0.0024	1.94E-11	0.0001	45
14	rs2239222	73011885	G	A	0.35	0.0189	0.0024	2.83E-15	0.0002	62
14	rs17580	94847262	A	T	0.05	0.0495	0.0053	5.19E-21	0.0003	88
14	rs11621792	24871926	T	C	0.45	-0.0350	0.0023	8.50E-53	0.0007	234
14	rs71413975	23723411	G	T	0.19	0.0248	0.0029	4.21E-18	0.0002	75
14	rs28929474	94844947	T	C	0.02	0.1941	0.0080	1.56E-128	0.0019	582
14	rs2494748	105258892	T	C	0.62	-0.0200	0.0023	1.26E-17	0.0002	73
14	rs13379043	74250126	C	T	0.28	0.0187	0.0026	4.30E-13	0.0002	53
15	rs17184382	63792486	C	A	0.43	0.0206	0.0023	2.01E-19	0.0003	81
15	rs4924339	35185603	G	A	0.73	-0.0160	0.0026	4.67E-10	0.0001	39
15	rs79391862	53739426	C	A	0.01	-0.1704	0.0098	5.65E-68	0.0010	304
15	rs139974673	44027885	C	T	0.03	-0.1109	0.0073	9.65E-53	0.0007	234
15	rs8025155	53093869	G	C	0.14	-0.0453	0.0033	5.24E-43	0.0006	189
15	rs2454352	36047091	C	T	0.24	0.0170	0.0027	2.70E-10	0.0001	40
15	rs12914895	51054195	G	C	0.49	0.0136	0.0023	2.66E-09	0.0001	35
15	rs76126845	75827909	T	G	0.06	0.0281	0.0049	7.25E-09	0.0001	33
15	rs60673924	41041105	AT	A	0.45	0.0133	0.0024	1.83E-08	0.0001	32
15	rs56332871	96714816	A	C	0.27	0.0663	0.0026	5.20E-148	0.0021	672
15	rs79240050	96667048	A	G	0.03	0.0562	0.0070	7.28E-16	0.0002	65
16	rs1558902	53803574	A	T	0.40	-0.0171	0.0023	1.26E-13	0.0002	55
16	rs55729432	88511548	G	C	0.27	0.0144	0.0026	2.60E-08	0.0001	31
16	rs56731455	88068078	AT	A	0.57	-0.0234	0.0023	3.33E-24	0.0003	103
16	rs37065	58571309	G	A	0.53	-0.0126	0.0023	2.64E-08	0.0001	31
16	rs11075016	11901557	G	A	0.28	0.0207	0.0025	2.17E-16	0.0002	67
16	rs12921195	4677604	A	C	0.14	-0.0185	0.0034	4.42E-08	0.0001	30
16	rs4782568	83980529	G	C	0.45	0.0253	0.0023	1.97E-28	0.0004	122

16	rs11644601	15172118	C	T	0.30	0.0219	0.0025	9.98E-19	0.0002	78
16	rs35280834	28423847	A	C	0.29	-0.0175	0.0027	1.15E-10	0.0001	42
17	rs78081080	7320581	T	C	0.08	-0.0784	0.0042	2.68E-76	0.0011	342
17	rs12952818	17988591	A	G	0.62	0.0280	0.0024	1.42E-32	0.0005	141
17	rs148920237	47293000	T	C	0.02	-0.0459	0.0075	8.10E-10	0.0001	38
17	rs369322961	47398246	T	G	0.09	0.1160	0.0040	4.19E-181	0.0026	825
17	rs7350906	45588112	A	G	0.51	0.0332	0.0023	1.90E-48	0.0083	214
17	rs1801689	64210580	C	A	0.03	-0.0787	0.0067	4.06E-32	0.0004	139
17	rs12943365	29680526	G	C	0.61	0.0266	0.0023	2.01E-30	0.0004	131
17	rs11657440	79480686	C	T	0.42	-0.0165	0.0023	7.35E-13	0.0002	51
17	rs7217661	65266562	C	T	0.52	-0.0151	0.0023	3.06E-11	0.0001	44
17	rs112220485	40522713	C	T	0.09	-0.0249	0.0040	7.37E-10	0.0001	38
17	rs10401031	73799110	C	A	0.25	0.0160	0.0028	8.83E-09	0.0001	33
17	rs858519	7531965	C	T	0.56	0.2045	0.0023	1.00E-200	0.0257	8220
17	rs12948480	73154247	A	G	0.65	-0.0236	0.0024	3.11E-23	0.0003	99
17	rs150365717	47289986	C	T	0.01	0.0694	0.0094	1.40E-13	0.0002	55
17	rs190408119	79989546	T	C	0.57	0.0135	0.0023	8.47E-09	0.0001	33
17	rs7220103	47221643	G	A	0.04	0.0594	0.0058	1.85E-24	0.0003	104
17	rs1050541	7560835	G	T	0.56	0.1228	0.0023	1.00E-200	0.0088	2759
17	rs7223885	7288085	T	C	0.10	-0.0853	0.0038	5.22E-110	0.0016	497
17	rs8077638	1640793	T	C	0.22	0.0352	0.0027	6.98E-38	0.0005	166
17	rs149854886	7865122	G	C	0.02	-0.0502	0.0086	5.27E-09	0.0001	34
18	rs4092465	55080437	G	A	0.65	0.0181	0.0024	3.86E-14	0.0002	57
18	rs7239714	71945255	A	G	0.69	0.0144	0.0024	3.65E-09	0.0001	35
18	rs6567160	57829135	C	T	0.23	-0.0208	0.0027	8.05E-15	0.0002	60
18	rs8087306	9094245	C	G	0.68	-0.0150	0.0024	7.14E-10	0.0001	38
19	rs4805881	33896432	C	A	0.67	0.0154	0.0024	1.30E-10	0.0001	41
19	rs12977524	19568244	G	A	0.18	-0.0189	0.0030	1.59E-10	0.0001	41
19	rs73038384	35510304	T	C	0.03	-0.0377	0.0065	5.26E-09	0.0001	34
19	rs35350976	59023174	G	A	0.18	0.0163	0.0030	3.26E-08	0.0001	31
19	rs202200760	17346854	C	G	0.04	0.1449	0.0064	3.51E-114	0.0017	516
19	rs45512696	35550878	T	C	0.18	0.0365	0.0030	2.08E-34	0.0005	150
19	rs10408163	47597102	C	T	0.72	-0.0148	0.0025	3.48E-09	0.0001	35
19	rs34514836	46385438	C	A	0.12	0.0525	0.0035	5.33E-51	0.0007	226
19	rs146009456	46397978	T	TTTTC	0.11	0.0526	0.0037	4.85E-45	0.0006	198
19	rs4804414	7223785	T	C	0.43	-0.0279	0.0023	3.20E-34	0.0005	149
19	rs8107967	7972615	G	A	0.57	0.0145	0.0023	2.47E-10	0.0001	40
19	rs2260414	2796316	A	G	0.29	0.0383	0.0025	7.79E-53	0.0007	234
19	rs273492	18237882	A	G	0.26	-0.0241	0.0026	1.49E-20	0.0003	86
19	rs111981233	50016479	G	T	0.08	0.0387	0.0042	2.28E-20	0.0003	86
20	rs6129802	39920014	T	C	0.21	0.0227	0.0028	2.20E-16	0.0002	67
20	rs6063848	51238004	T	G	0.65	-0.0151	0.0024	3.80E-10	0.0001	39

20	rs6073431	43040569	T	C	0.53	0.0342	0.0023	1.11E-49	0.0007	220
20	rs34587839	32300671	A	G	0.15	-0.0234	0.0031	9.50E-14	0.0002	55
20	rs2618566	17844684	T	G	0.66	-0.0147	0.0024	7.66E-10	0.0001	38
20	rs4810580	45594295	G	T	0.22	-0.0159	0.0028	9.37E-09	0.0001	33
20	rs55987409	49569025	T	C	0.07	0.0270	0.0044	8.32E-10	0.0001	38
20	rs6120663	33081906	A	C	0.44	-0.0176	0.0023	1.24E-14	0.0002	59
20	rs112756706	62362850	T	A	0.24	0.0190	0.0027	1.90E-12	0.0002	50
21	rs9980890	40715947	G	A	0.47	0.0127	0.0023	2.19E-08	0.0001	31
22	rs6005837	29090481	C	G	0.66	-0.0254	0.0024	4.68E-26	0.0004	111
22	rs757869	30764453	A	G	0.70	-0.0272	0.0025	5.52E-28	0.0004	120
22	rs4820091	21940189	G	T	0.18	0.0235	0.0030	2.04E-15	0.0002	63
22	rs138699	39129708	A	G	0.25	-0.0146	0.0026	3.30E-08	0.0001	31
22	rs738409	44324727	G	C	0.22	0.0443	0.0027	2.24E-58	0.0008	260
22	rs6003989	24295496	A	G	0.32	0.0153	0.0025	4.73E-10	0.0001	39

Due to 23 SNPs (rs71514237, rs6603985, rs79094524, rs72948328, rs71401561, rs34261514, rs113986045, rs35694679, rs371744786, rs76120024, rs113976091, rs10750766, rs7306128, rs10841521, rs36179992, rs8025155, rs60673924, rs56731455, rs12921195, rs146009456, rs138699, rs6003989) in linkage disequilibrium ($r^2 > 0.001$) were removed, there were 241 instrument SNPs were included to build genetic risk score for MR analyses.

Abbreviation: SNP, single-nucleotide polymorphism; EAF, effect allele frequency; SE, standard error.

Supplementary Table S4 Mendelian randomization and sensitivity analyses between sex hormone-binding globulin levels and stroke risk

**Stroke subtypes**	**Sample size**	**Cases**	**Final IVs[a]**	**OR (95%CI)**	**GRS *P* value[b]**	**Cochran Q *P* value[c]**	**IVW I^2 (%)[c]**	**Egger intercept *P* value[c]**
AS	446,696	40,585	230	0.941 (0.898, 0.984)	0.005	4.38E-03	20.8	0.388
AIS	440,328	34,217	221	0.951 (0.922, 0.981)	0.013	1.95E-04	24.6	0.316
CES	211,763	7,193	233	0.991 (0.903, 1.079)	0.838	0.104	10.6	0.103
LAS	150,765	4,373	229	0.932 (0.819, 1.046)	0.227	0.090	11.3	0.623
SVS	198,048	5,386	238	0.871 (0.765, 0.977)	0.010	0.028	15.7	0.791
ICH	3,026	1,545	114	0.810 (0.473, 1.147)	0.219	0.032	20.7	0.084

[a] After removing any potential pleiotropic and outlier SNPs for the exposure, along with the SNPs were not available in the outcome summary statistics, the final SNPs were included to build genetic risk score (GRS) for MR analyses.

[b] The MR result is considered statistically significant when $P < 0.05$.

[c] There is no significant heterogeneity when $I^2 < 25\%$ or Cochran Q-derived $P > 0.05$ detected by inverse variance-weighted (IVW) method, and $P > 0.05$ of the MR Egger intercept test indicates no directional pleiotropic effect.

Abbreviation: IVs, instrument variables; OR, odds ratio; CI, confidence interval; AS, any stroke; AIS, any ischemic stroke; CES, cardioembolic stroke; LAS, large-artery stroke; SVS, small vessel stroke; ICH, intracerebral hemorrhage.

Supplementary Table S5 Mendelian randomization and sensitivity analyses between sex hormone-binding globulin levels and cardiometabolic traits in the discovery datasets

Outcomes	Final IVs[a]	OR/β (95%CI)	GRS *P* value[b]	Cochran Q *P* value[c]	IVW I^2 (%)[c]	Egger intercept *P* value[c]	IVW-mre *P* value[d]
BMI	98	-0.058 (-0.093, -0.023)	0.001	0.130	14.0	0.316	NA
WC	92	-0.091 (-0.136, -0.046)	5.85E-05	0.004	30.3	0.578	7.95E-04
WHR	88	-0.057 (-0.084, -0.030)	4.83E-05	9.47E-04	35.0	0.987	0.001
FG	150	-0.034 (-0.054, -0.014)	0.007	0.013	21.5	0.785	NA
FI	133	-0.028 (-0.046, -0.010)	0.002	3.53E-03	26.6	0.116	0.007
HbA1c	109	0.020 (0.010, 0.030)	1.31E-05	2.27E-08	48.7	0.683	1.71E-03
T2DM	48	0.684 (0.400, 0.968)	4.77E-03	0.294	9.2	0.057	NA
TC	89	-0.023 (-0.080, 0.034)	0.221	8.91E-06	43.9	0.772	0.359
TG	66	-0.188 (-0.249, -0.127)	2.22E-09	1.68E-05	47.3	0.797	1.42E-05
LDL-C	70	-0.027 (-0.068, 0.014)	0.201	2.24E-04	41.5	0.162	0.328
HDL-C	71	0.141 (0.094, 0.188)	5.64E-09	3.60E-04	40.2	0.245	6.62E-06
SBP	88	-0.799 (-1.068, -0.530)	5.24E-09	1.26E-08	52.1	0.229	5.28E-05
DBP	81	-0.436 (-0.605, -0.267)	3.51E-07	3.51E-07	54.7	0.450	6.05E-04
Hypertension	237	1.000 (0.999, 1.000)	0.857	0.240	5.9	0.285	NA
Adiponectin	88	0.037 (0.008, 0.066)	0.014	1.09E-03	34.6	0.658	0.047

[a] After removing any potential pleiotropic and outlier SNPs for the exposure, along with the SNPs were not available in the outcome summary statistics, the final SNPs were included to build genetic risk score (GRS) for MR analyses.

[b] The MR result is considered statistically significant when $P < 0.05$.

[c] There is no significant heterogeneity when $I^2 < 25\%$ or Cochran Q-derived $P > 0.05$ detected by inverse variance-weighted (IVW) method, and $P > 0.05$ of the MR Egger intercept test indicates no directional pleiotropic effect.

[d] If heterogeneity still exists after dealing with conventional sensitivity analyses, the multiplicative random effects of the IVW (IVW-mre) method would be further used as a complementary analysis to determine the causal relationship. The MR result is considered statistically significant when IVW-mre $P < 0.05$.

Abbreviation: IVs, instrument variables; OR, odds ratio; CI, confidence interval; BMI, body mass index; WHR, waist-to-hip ratio; WC, waist circumference; FG, fasting glucose; FS, fasting insulin; HbA1c, glycosylated hemoglobin; T2DM, type 2 diabetes mellitus; TC, total cholesterol; TG, triglyceride; LDL-C, low-density lipoprotein cholesterol; HDL-C, high-density lipoprotein cholesterol; SBP, systolic blood pressure; DBP, diastolic blood pressure; NA, not available.

Supplementary Table S6 Mendelian randomization and sensitivity analyses between sex hormone-binding globulin levels

and cardiometabolic traits in the replication datasets

Outcomes	Final IVs[a]	OR/β (95%CI)	GRS *P* value[b]	Cochran Q *P* value[c]	IVW I^2 (%)[c]	Egger intercept *P* value[c]	IVW-mre *P* value[d]
BMI	104	-0.005 (-0.046, 0.036)	0.820	2.56E-04	35.8	0.592	0.856
WC	119	-0.039 (-0.057, -0.021)	2.04E-05	4.52E-10	48.4	0.060	2.68E-03
WHR	189	-0.065 (-0.094, -0.036)	2.56E-05	2.79E-10	43.5	0.249	1.59E-03
FG	187	-0.029 (-0.049, -0.009)	0.006	0.006	22.0	0.491	NA
FI	184	-0.017 (-0.039, 0.005)	0.123	0.033	16.7	0.600	NA
HbA1c	80	-0.040 (-0.095, 0.015)	0.162	7.99E-04	36.7	0.068	0.266
T2DM	33	0.834 (0.749, 0.918)	2.27E-05	4.41E-04	51.1	0.310	0.003
TG	183	-0.131 (-0.213, -0.049)	0.002	2.38E-03	24.3	0.192	NA
HDL-C	112	0.103 (-0.024, 0.230)	0.113	0.148	12.3	0.236	0.325
SBP	104	-0.055 (-0.075, -0.035)	1.70E-08	4.46E-10	52.4	0.867	1.00E-04
DBP	168	-0.027 (-0.041, -0.013)	9.37E-05	1.64E-12	55.6	0.962	0.001
Adiponectin	77	0.207 (-0.020, 0.434)	0.075	0.238	10.0	0.770	0.091

[a] After removing any potential pleiotropic and outlier SNPs for the exposure, along with the SNPs were not available in the outcome summary statistics, the final SNPs were included to build genetic risk score (GRS) for MR analyses.

[b] The MR result is considered statistically significant when $P < 0.05$.

[c] There is no significant heterogeneity when $I^2 < 25\%$ or Cochran Q-derived $P > 0.05$ detected by inverse variance-weighted (IVW) method, and $P > 0.05$ of the MR Egger intercept test indicates no directional pleiotropic effect.

[d] If heterogeneity still exists after dealing with conventional sensitivity analyses, the multiplicative random effects of the IVW (IVW-mre) method would be further used as a complementary analysis to determine the causal relationship. The MR result is considered statistically significant when IVW-mre $P < 0.05$.

Abbreviation: IVs, instrument variables; OR, odds ratio; CI, confidence interval; BMI, body mass index; WC, waist circumference; WHR, waist-to-hip ratio; FG, fasting glucose; FS, fasting insulin; HbA1c, glycosylated hemoglobin; T2DM, type 2 diabetes mellitus; TG, triglyceride; HDL-C, high-density lipoprotein cholesterol; SBP, systolic blood pressure; DBP, diastolic blood pressure; NA, not available.

Supplementary Table S7 Mendelian randomization and sensitivity analyses between the potential cardiometabolic mediators and any stroke

Outcomes	Total IVs	Variance explained (R^2, %)	F statistic for the SNP-GRS	F statistic for each SNP, median (range)	Final IVs[a]	OR (95%CI)	GRS *P* value[b]	Cochran Q *P* value[c]	IVW I^2 (%)[c]	Egger intercept *P* value[c]	IVW-mre *P* value[d]
WC	42	1.1	56	38 (29, 447)	39	1.135 (1.016, 1.255)	0.037	0.007	39.5	0.505	0.010
WHR	38	1.0	56	46 (28, 153)	36	1.213 (1.095, 1.330)	0.001	0.001	48.1	0.760	0.021
FG	14	2.8	85	46 (31, 335)	10	0.989 (0.842, 1.136)	0.884	0.257	20.2	0.821	NA
T2DM	13	1.2	64	39 (26, 286)	10	1.028 (0.983, 1.073)	0.225	0.242	21.9	0.643	NA
TG	54	9.2	181	59 (30, 1258)	54	1.008 (0.949, 1.073)	0.788	0.052	25.1	0.146	NA
SBP	461	4.8	83	50 (30, 628)	331	1.033 (1.029, 1.036)	7.82E-54	3.96E-05	25.2	0.353	1.05E-40
DBP	460	4.9	85	51 (30, 816)	419	1.048 (1.042, 1.054)	1.93E-56	2.37E-07	28.0	0.454	3.71E-41

[a] After removing any potential pleiotropic and outlier SNPs for the exposure, along with the SNPs were not available in the outcome summary statistics, the final SNPs were included to build genetic risk score (GRS) for MR analyses.

[b] The MR result is considered statistically significant when $P < 0.05$.

[c] There is no significant heterogeneity when $I^2 < 25\%$ or Cochran Q-derived $P > 0.05$ detected by inverse variance-weighted (IVW) method, and $P > 0.05$ from the MR Egger intercept test indicates no directional pleiotropic effect.

[d] If heterogeneity still exists after dealing with conventional sensitivity analyses, the multiplicative random effects of the IVW (IVW-mre) method would be further used as a complementary analysis to determine the causal relationship. The MR result is considered statistically significant when IVW-mre $P < 0.05$.

Abbreviation: IVs, instrument variables; OR, odds ratio; CI, confidence interval; WC, waist circumference; WHR, waist-to-hip ratio; FG, fasting glucose; T2DM, type 2 diabetes mellitus; TG, triglyceride; SBP, systolic blood pressure; DBP, diastolic blood pressure; NA, not available.

Supplementary Table S8 Mendelian randomization and sensitivity analyses between the potential cardiometabolic mediators and any ischemic stroke

Outcomes	Total IVs	Variance explained (R^2, %)	F statistic for the SNP-GRS	F statistic for each SNP, median (range)	Final IVs[a]	OR (95%CI)	GRS *P* value[b]	Cochran Q *P* value[c]	IVW I^2 (%)[c]	Egger intercept *P* value[c]	IVW-mre *P* value[d]
WC	42	1.1	56	38 (29, 447)	37	1.236 (1.122, 1.350)	2.36E-04	0.084	25.3	0.836	NA
WHR	38	1.0	56	46 (28, 153)	35	1.154 (1.042, 1.265)	0.012	0.025	34.6	0.773	0.042
FG	14	2.8	85	46 (31, 335)	10	1.024 (0.885, 1.163)	0.736	0.097	39.2	0.467	NA
T2DM	13	1.2	64	39 (26, 286)	10	1.048 (1.005, 1.091)	0.030	0.481	0.0	0.310	NA
TG	54	9.2	181	59 (30, 1258)	52	1.018 (0.965, 1.071)	0.504	0.213	13.2	0.165	NA
SBP	461	4.8	83	50 (30, 628)	318	1.034 (1.030, 1.037)	2.21E-61	5.69E-09	34.3	0.207	6.39E-35
DBP	460	4.9	85	51 (30, 816)	407	1.050 (1.044, 1.056)	6.11E-69	2.98E-09	31.7	0.238	1.13E-47

[a] After removing any potential pleiotropic and outlier SNPs for the exposure, along with the SNPs were not available in the outcome summary statistics, the final SNPs were included to build genetic risk score (GRS) for MR analyses.

[b] The MR result is considered statistically significant when $P < 0.05$.

[c] There is no significant heterogeneity when $I^2 < 25\%$ or Cochran Q-derived $P > 0.05$ detected by inverse variance-weighted (IVW) method, and $P > 0.05$ from the MR Egger intercept test indicates no directional pleiotropic effect.

[d] If heterogeneity still exists after dealing with conventional sensitivity analyses, the multiplicative random effects of the IVW (IVW-mre) method would be further used as a complementary analysis to determine the causal relationship. The MR result is considered statistically significant when IVW-mre $P < 0.05$.

Abbreviation: IVs, instrument variables; OR, odds ratio; CI, confidence interval; WC, waist circumference; WHR, waist-to-hip ratio; FG, fasting glucose; T2DM, type 2 diabetes mellitus; TG, triglyceride; SBP, systolic blood pressure; DBP, diastolic blood pressure; NA, not available.

Supplementary Table S9 Mendelian randomization and sensitivity analyses between the potential cardiometabolic mediators and small vessel stroke

Outcomes	Total IVs	Variance explained (R^2, %)	F statistic for the SNP-GRS	F statistic for each SNP, median (range)	Final IVs[a]	OR (95%CI)	GRS *P* value[b]	Cochran Q *P* value[c]	IVW I^2 (%)[c]	Egger intercept *P* value[c]	IVW-mre *P* value[d]
WC	42	1.1	56	38 (29, 447)	39	0.810 (0.510, 1.110)	0.169	0.004	42.1	0.861	0.295
WHR	38	1.0	56	46 (28, 153)	36	1.402 (1.104, 1.700)	0.026	0.056	28.8	0.669	NA
FG	14	2.8	85	46 (31, 335)	10	1.064 (0.697, 1.430)	0.742	0.225	23.7	0.535	NA
T2DM	13	1.2	64	39 (26, 286)	10	1.085 (0.972, 1.199)	0.157	0.461	0.0	0.757	NA
TG	54	9.2	181	59 (30, 1258)	54	1.182 (1.035, 1.329)	0.026	0.147	16.9	0.141	NA
SBP	461	4.8	83	50 (30, 628)	331	1.046 (1.036, 1.056)	1.59E-18	2.91E-04	22.5	0.645	NA
DBP	460	4.9	85	51 (30, 816)	425	1.074 (1.060, 1.087)	4.30E-22	7.03E-04	19.0	0.429	NA

[a] After removing any potential pleiotropic and outlier SNPs for the exposure, along with the SNPs were not available in the outcome summary statistics, the final SNPs were included to build genetic risk score (GRS) for MR analyses.

[b] The MR result is considered statistically significant when $P < 0.05$.

[c] There is no significant heterogeneity when $I^2 < 25\%$ or Cochran Q-derived $P > 0.05$ detected by inverse variance-weighted (IVW) method, and $P > 0.05$ from the MR Egger intercept test indicates no directional pleiotropic effect.

[d] If heterogeneity still exists after dealing with conventional sensitivity analyses, the multiplicative random effects of the IVW (IVW-mre) method would be further used as a complementary analysis to determine the causal relationship. The MR result is considered statistically significant when IVW-mre $P < 0.05$.

Abbreviation: IVs, instrument variables; OR, odds ratio; CI, confidence interval; WC, waist circumference; WHR, waist-to-hip ratio; FG, fasting glucose; T2DM, type 2 diabetes mellitus; TG, triglyceride; SBP, systolic blood pressure; DBP, diastolic blood pressure; NA, not available.

Supplementary Table S10 Mendelian randomization and sensitivity analyses between the cardiometabolic mediators and sex hormone-binding globulin levels

Exposures	Total IVs	Final IVs[a]	MR analysis		Sensitivity analyses			
			β (95%CI)	GRS *P* value[b]	Cochran Q *P* value[c]	IVW I^2 (%)[c]	Egger intercept *P* value[c]	IVW-mre *P* value[d]
WC	42	23	-0.161 (-0.200, -0.122)	3.82E-16	0.016	42.1	0.268	7.34E-10
WHR	38	18	-0.144 (-0.189, -0.099)	3.56E-10	7.35E-04	59.3	0.495	6.24E-05
T2DM	13	4	-0.071 (-0.091, -0.051)	4.27E-13	0.520	0.0	0.860	NA
TG	54	12	-0.152 (-0.189, -0.115)	7.14E-16	0.049	44.2	0.727	1.67E-09
SBP	461	285	-0.003 (-0.005, -0.002)	1.03E-05	1.68E-15	44.8	0.064	0.001
DBP	460	368	-0.002 (-0.004, 0.000)	0.070	4.67E-33	53.5	0.434	0.070

[a] After removing any potential pleiotropic and outlier SNPs for the exposure, along with the SNPs were not available in the outcome summary statistics, the final SNPs were included to build genetic risk score (GRS) for MR analyses.

[b] The MR result is considered statistically significant when $P < 0.05$.

[c] There is no significant heterogeneity when $I^2 < 25\%$ or Cochran Q-derived $P > 0.05$ detected by inverse variance-weighted (IVW) method, and $P > 0.05$ from the MR Egger intercept test indicates no directional pleiotropic effect.

[d] If heterogeneity still exists after dealing with conventional sensitivity analyses, the multiplicative random effects of the IVW (IVW-mre) method would be further used as a complementary analysis to determine the causal relationship. The MR result was considered statistically significant when IVW-mre $P < 0.05$.

Abbreviation: IVs, instrument variables; CI, confidence interval; WC, waist circumference; WHR, waist-to-hip ratio; T2DM, type 2 diabetes mellitus; TG, triglyceride; SBP, systolic blood pressure; DBP, diastolic blood pressure; NA, not available.

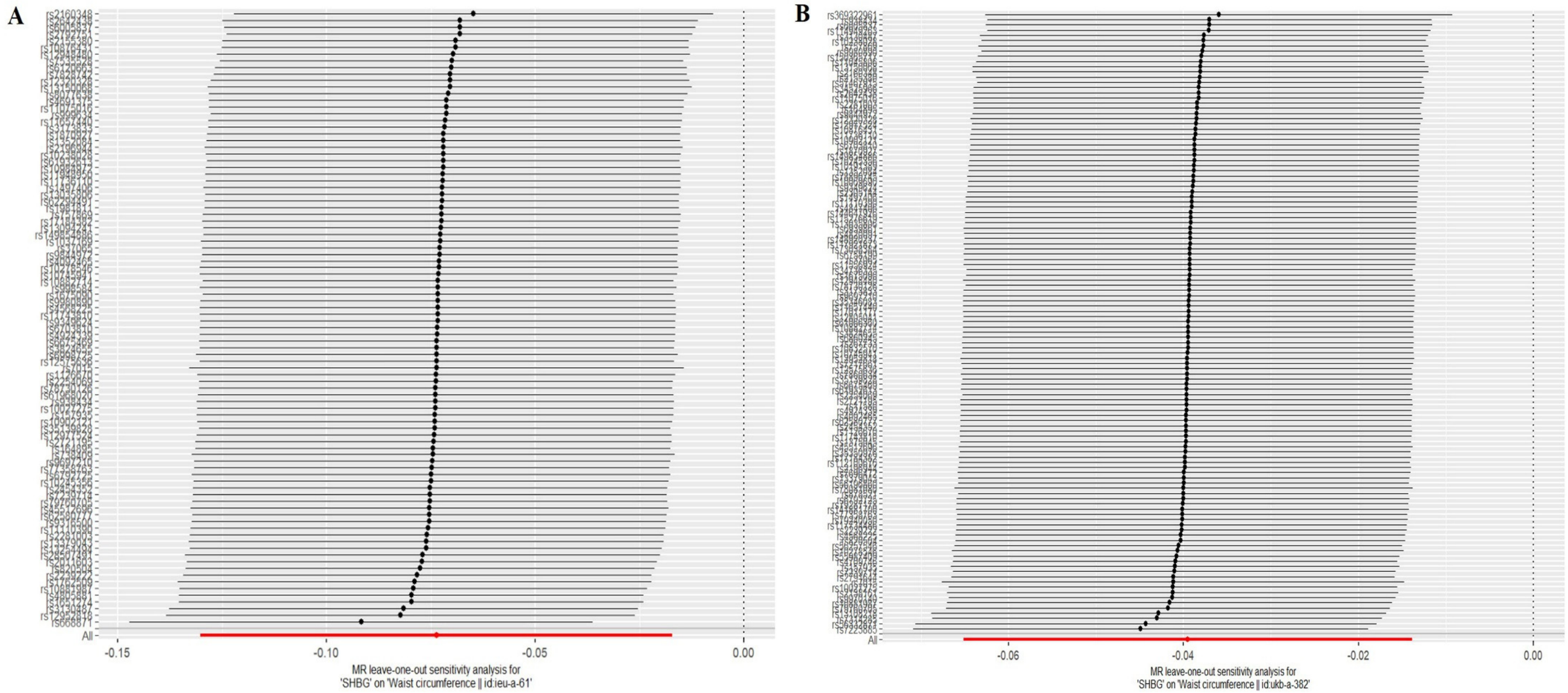

Supplementary Figure S1 Leave-one-out analysis for the causal estimates of sex hormone-binding globulin and waist circumference in the discovery dataset (A) and replication dataset (B)

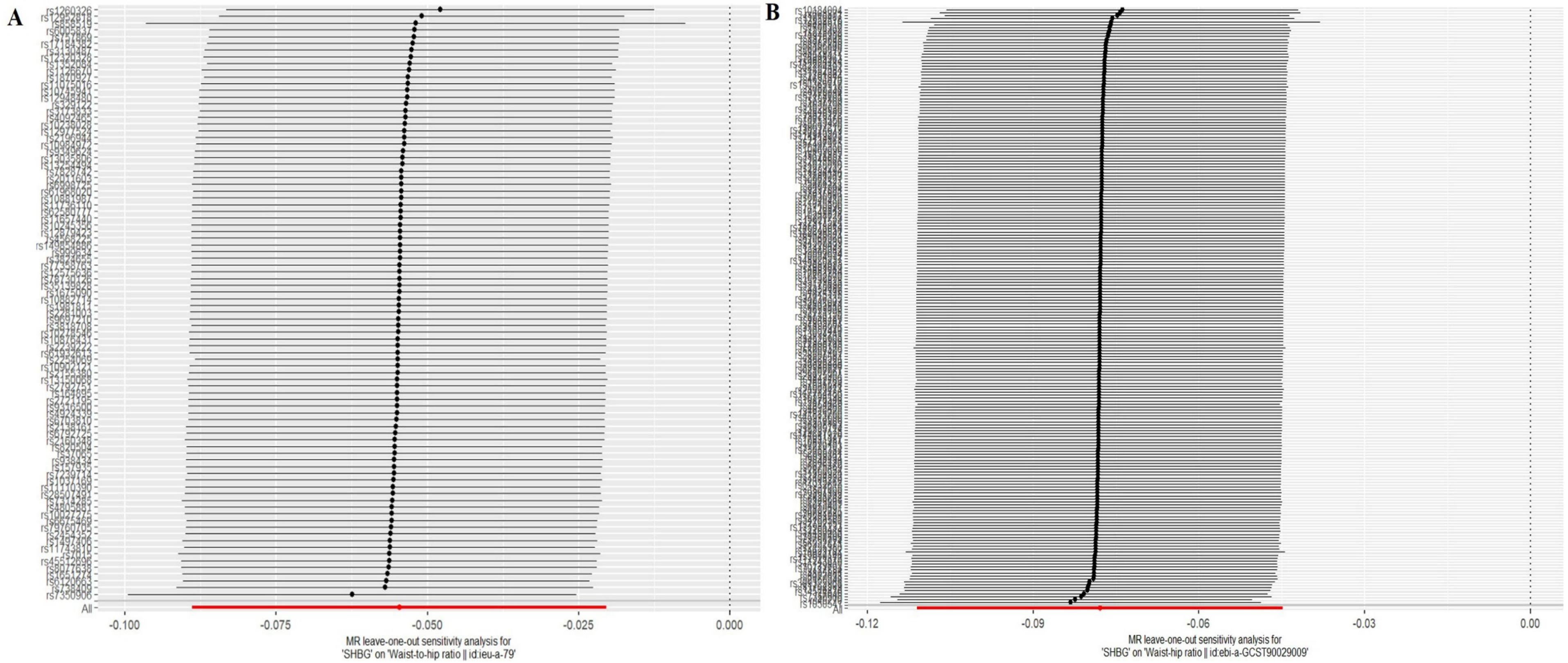

Supplementary Figure S2 Leave-one-out analysis for the causal estimates of sex hormone-binding globulin and waist-to-hip ratio in the discovery dataset (A) and replication dataset (B)

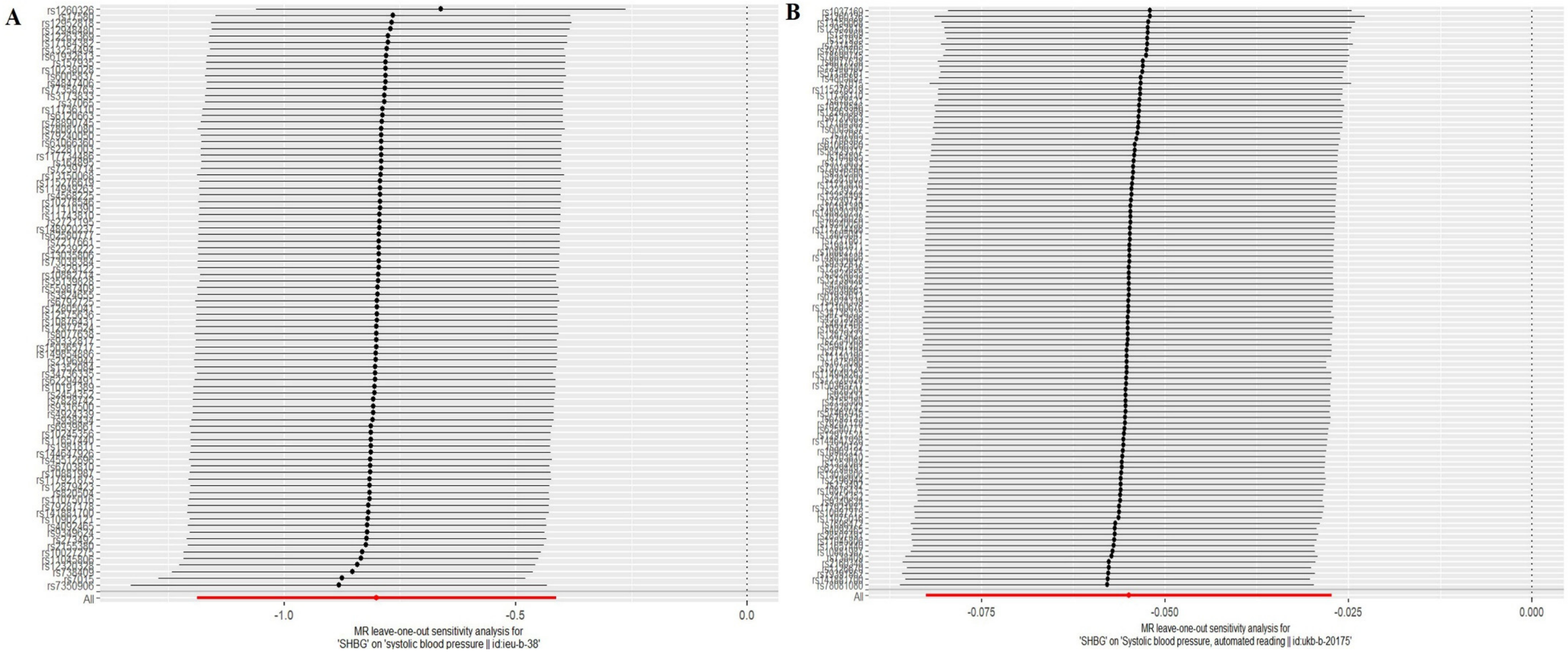

Supplementary Figure S3 Leave-one-out analysis for the causal estimates of sex hormone-binding globulin and systolic blood pressure in the discovery dataset (A) and replication dataset (B)

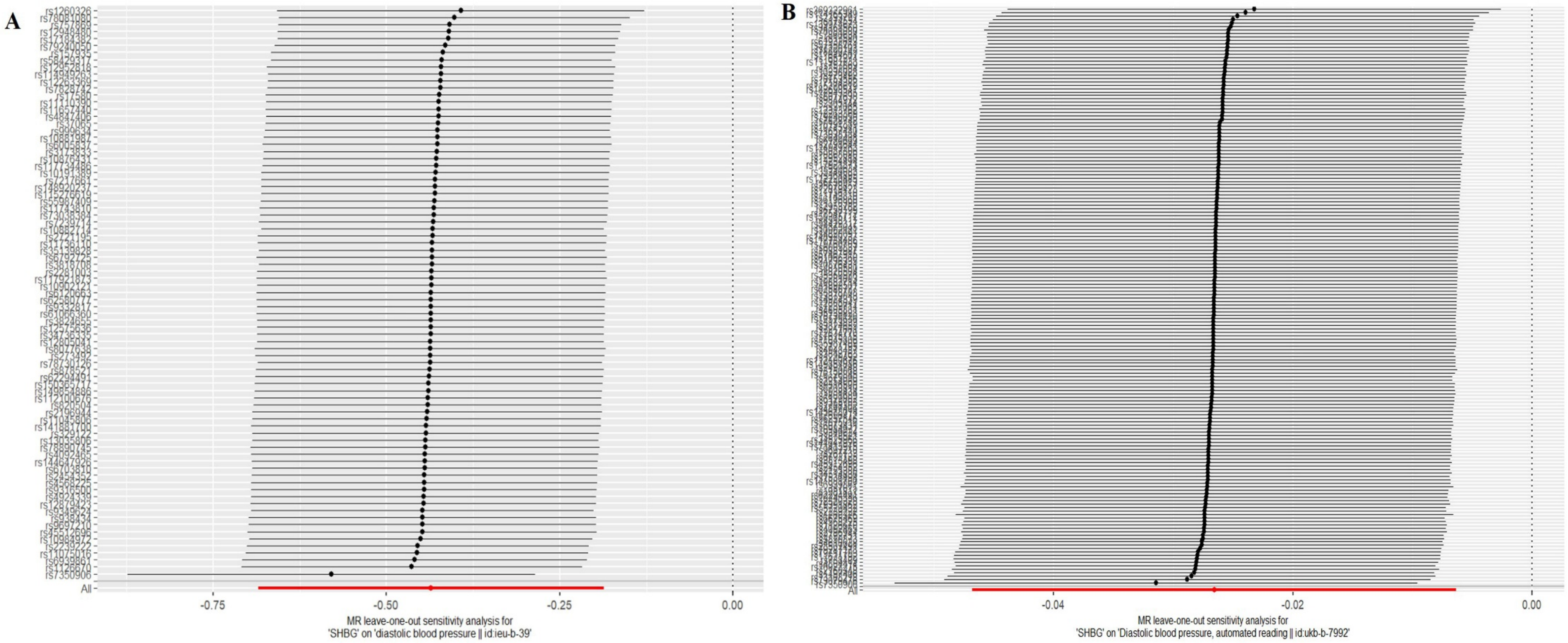

Supplementary Figure S4 Leave-one-out analysis for the causal estimates of sex hormone-binding globulin and diastolic blood pressure in the discovery dataset (A) and replication dataset (B)

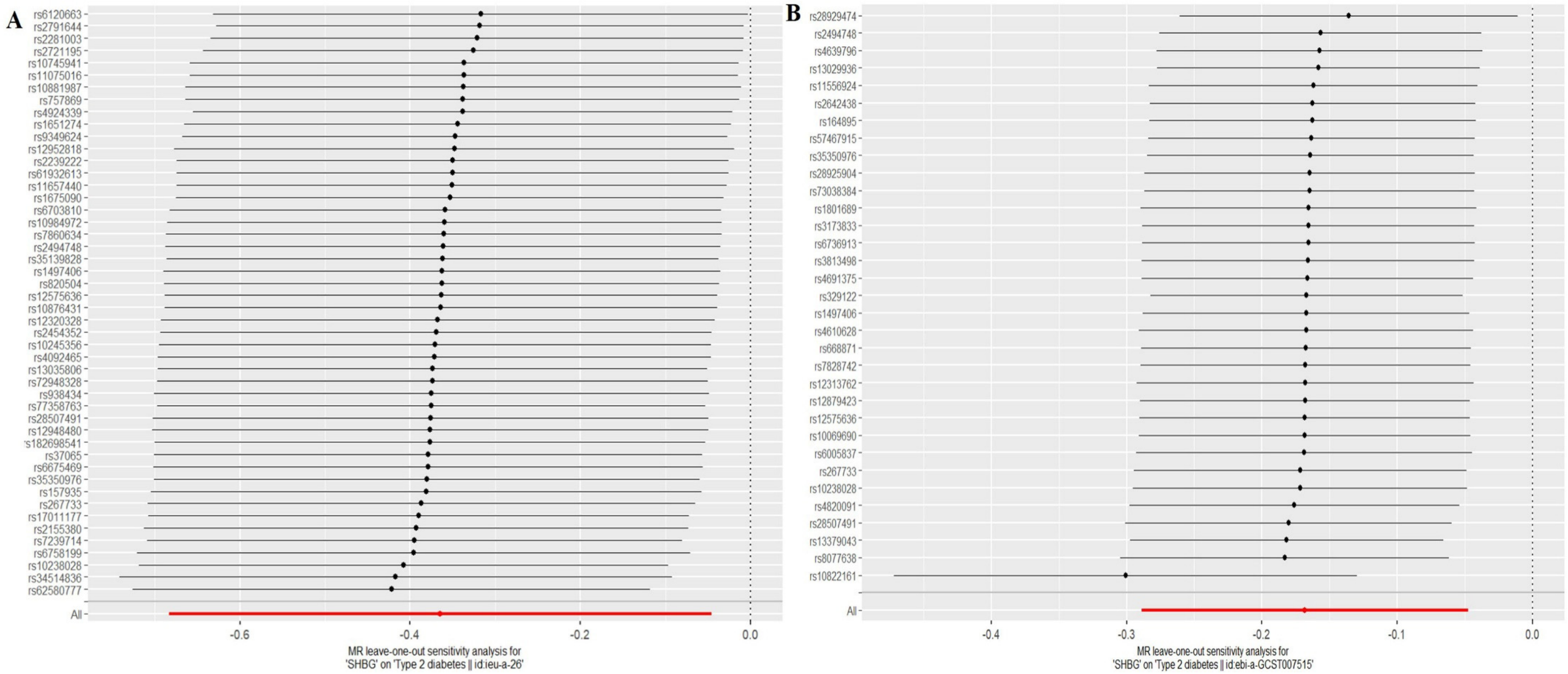

Supplementary Figure S5 Leave-one-out analysis for the causal estimates of sex hormone-binding globulin and type 2 diabetes mellitus in the discovery dataset (A) and replication dataset (B)

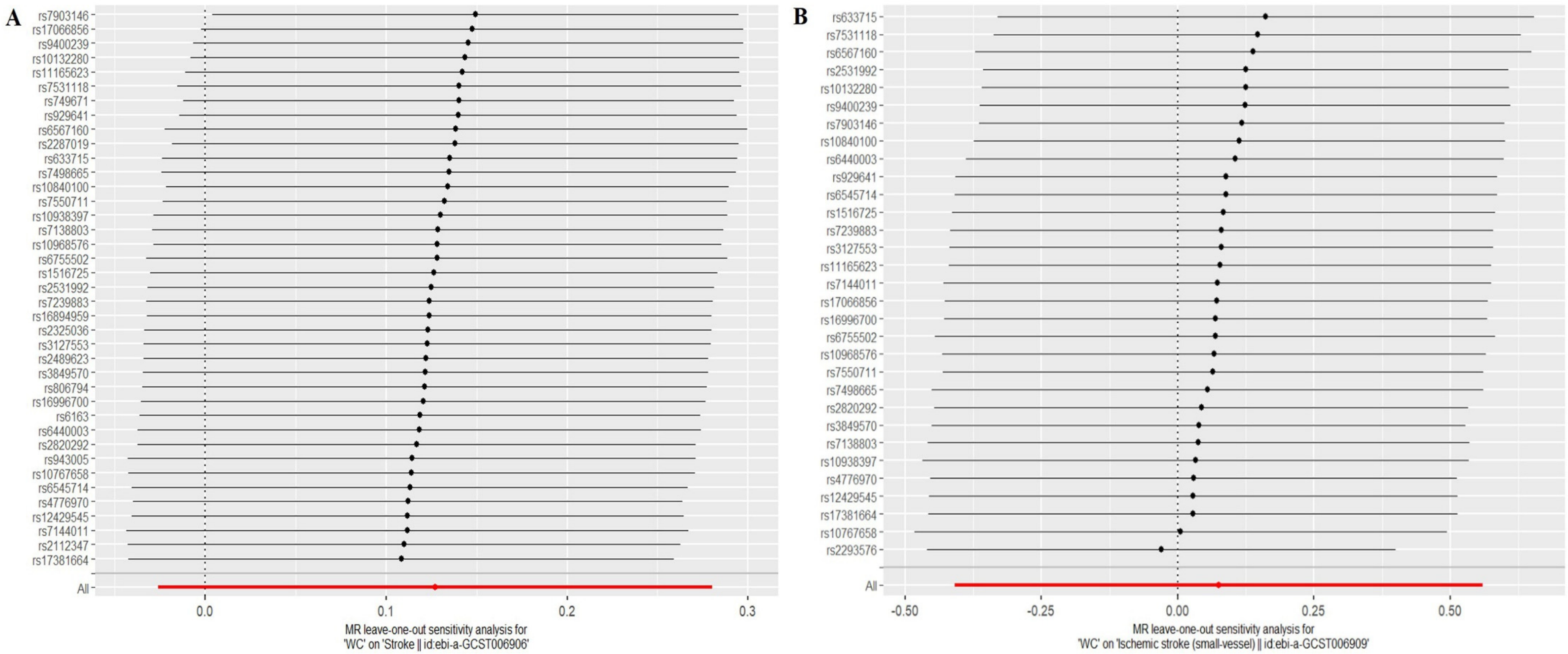

Supplementary Figure S6 Leave-one-out analysis for the causal estimates of waist circumference with any stroke (A) and small-vessel stroke (B)

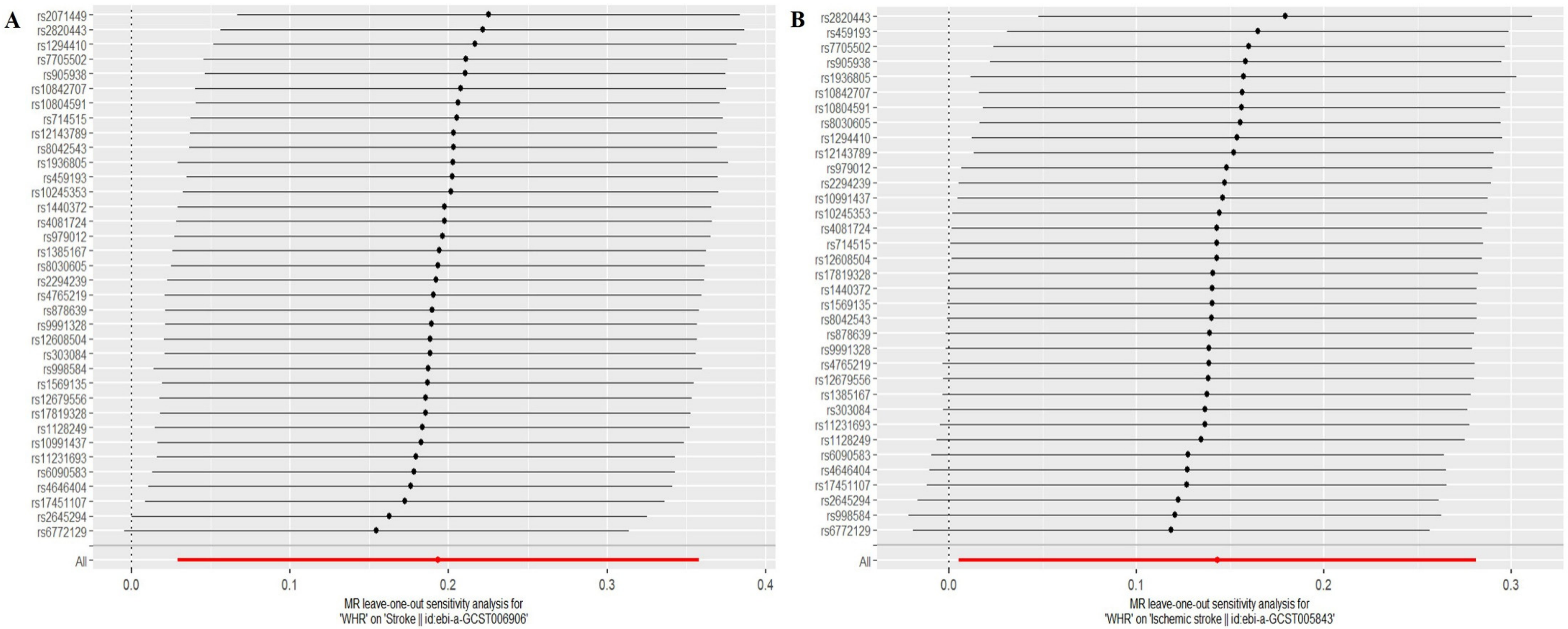

Supplementary Figure S7 Leave-one-out analysis for the causal estimates of waist-to-hip ratio with any stroke (A) and any ischemic stroke (B)

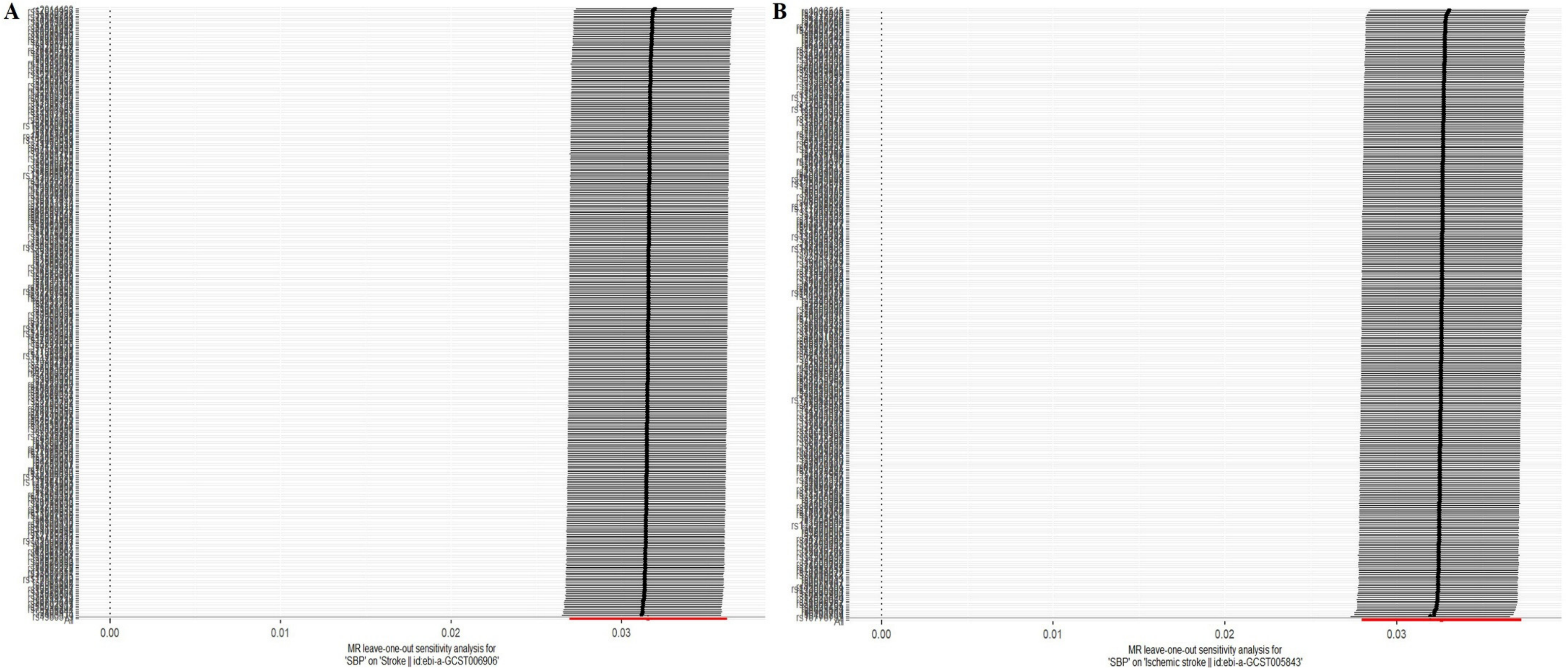

Supplementary Figure S8 Leave-one-out analysis for the causal estimates of systolic blood pressure with any stroke (A) and any ischemic stroke (B)

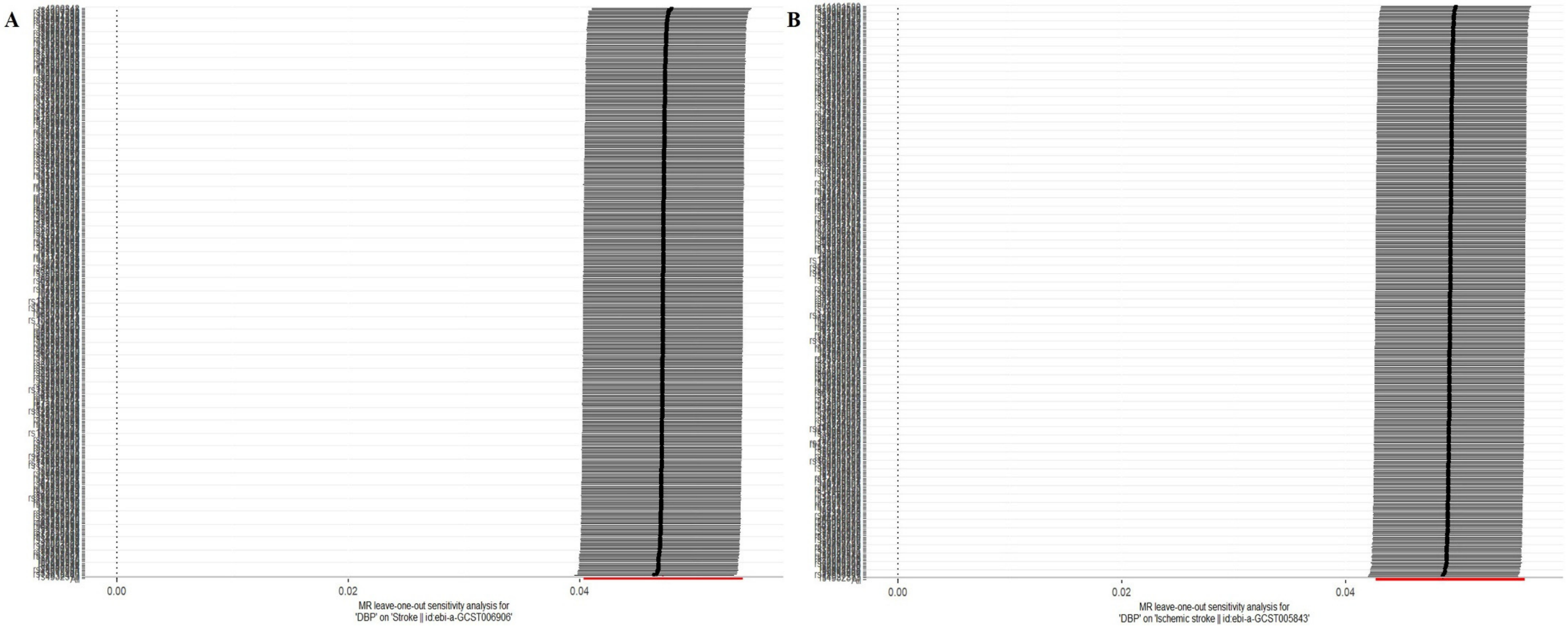

Supplementary Figure S9 Leave-one-out analysis for the causal estimates of diastolic blood pressure with any stroke (A) and any ischemic stroke (B)

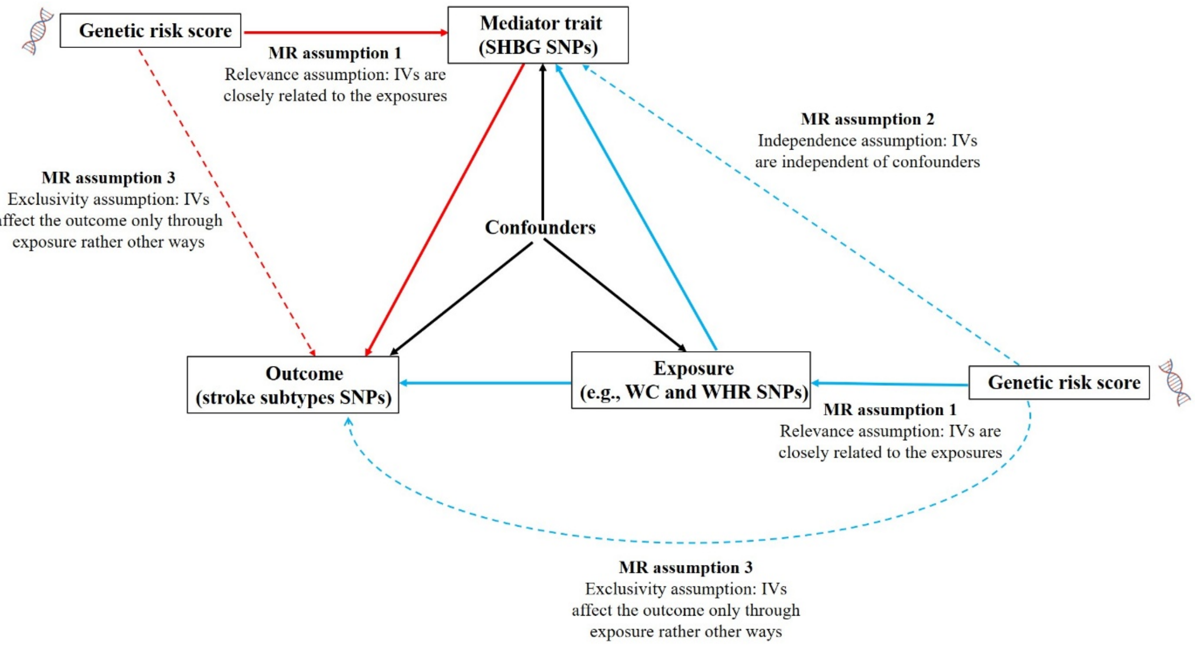

Supplementary Figure S10 Study design of the causal effects of the cardiometabolic mediators on stroke risk via sex hormone-binding globulin (SHBG). Herein, we constructed the genetic risk score (GRS) of the significant SNPs related to the exposure as instrument variables (IVs), and performed three two-sample Mendelian randomization (MR) tests to estimate the causal associations of exposure-outcome, exposure-mediator, and mediator-outcome. MR analysis should adhere to three core assumptions, assumption 1: the IVs are strongly associated with the exposure of interest (e.g., WC and WHR); assumption 2: the IVs should be independent of any confounders which correlated with exposure or outcome; assumption 3: the IVs affect the outcome only through the exposure rather than any alternative pathways. WC, waist circumference; WHR, waist-to-hip ratio.

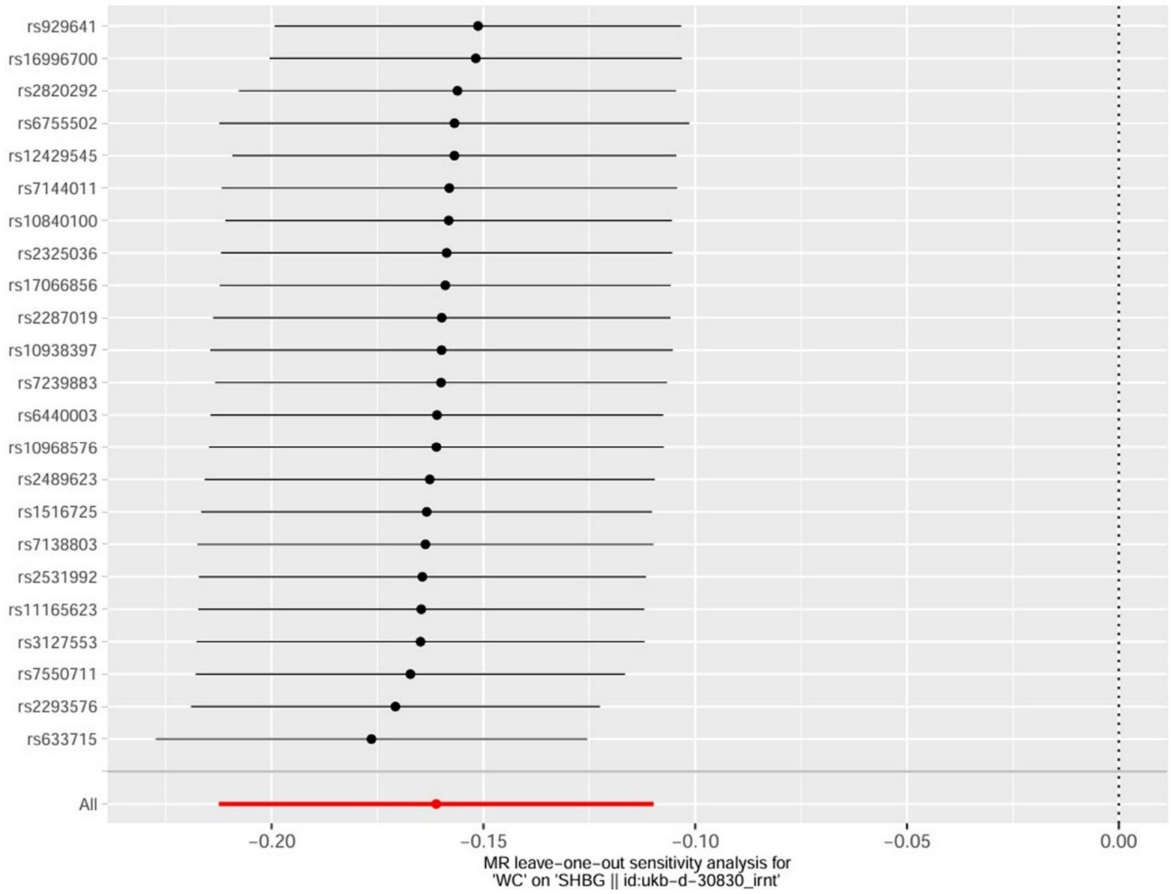

Supplementary Figure S11 Leave-one-out analysis for the causal estimates of waist circumference and sex hormone-binding globulin

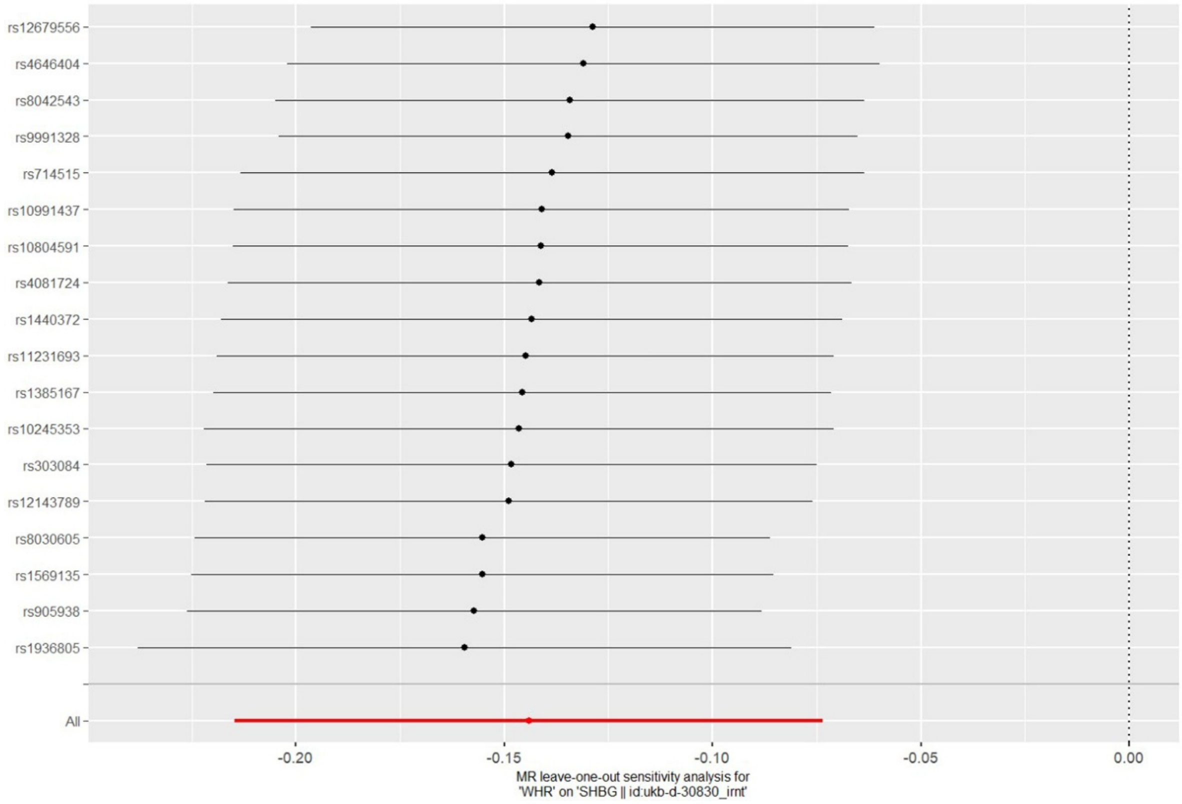

Supplementary Figure S12 Leave-one-out analysis for the causal estimates of waist-to-hip ratio and sex hormone-binding globulin

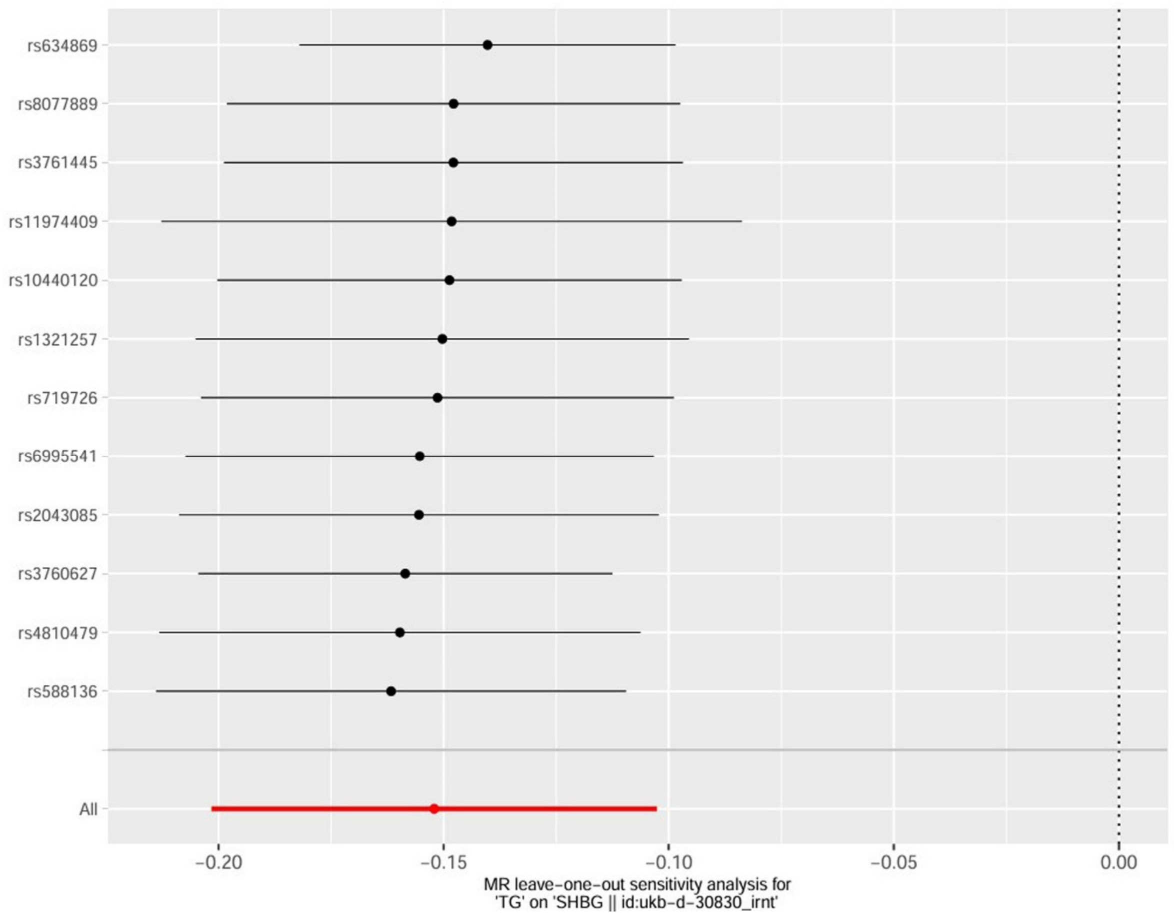

Supplementary Figure S13 Leave-one-out analysis for the causal estimates of triglyceride and sex hormone-binding globulin

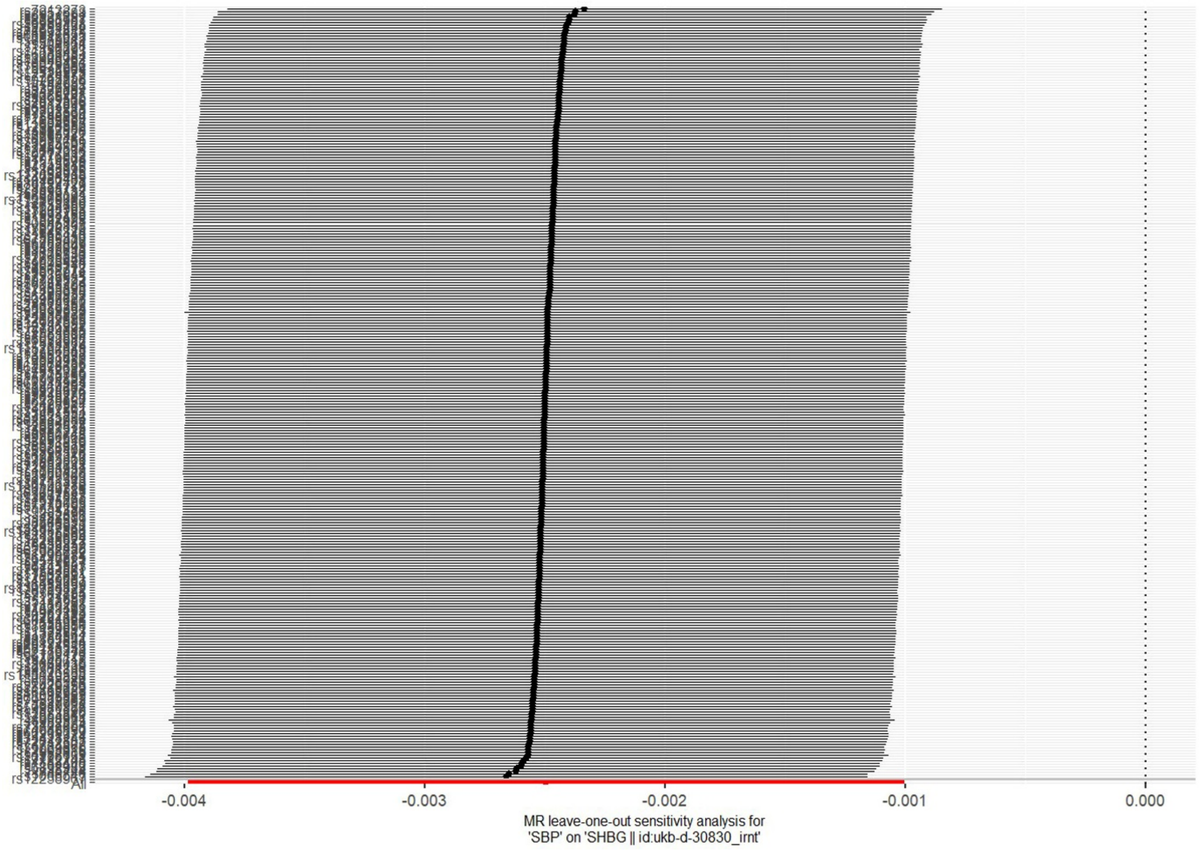

Supplementary Figure S14 Leave-one-out analysis for the causal estimates of systolic blood pressure and sex hormone-binding globulin

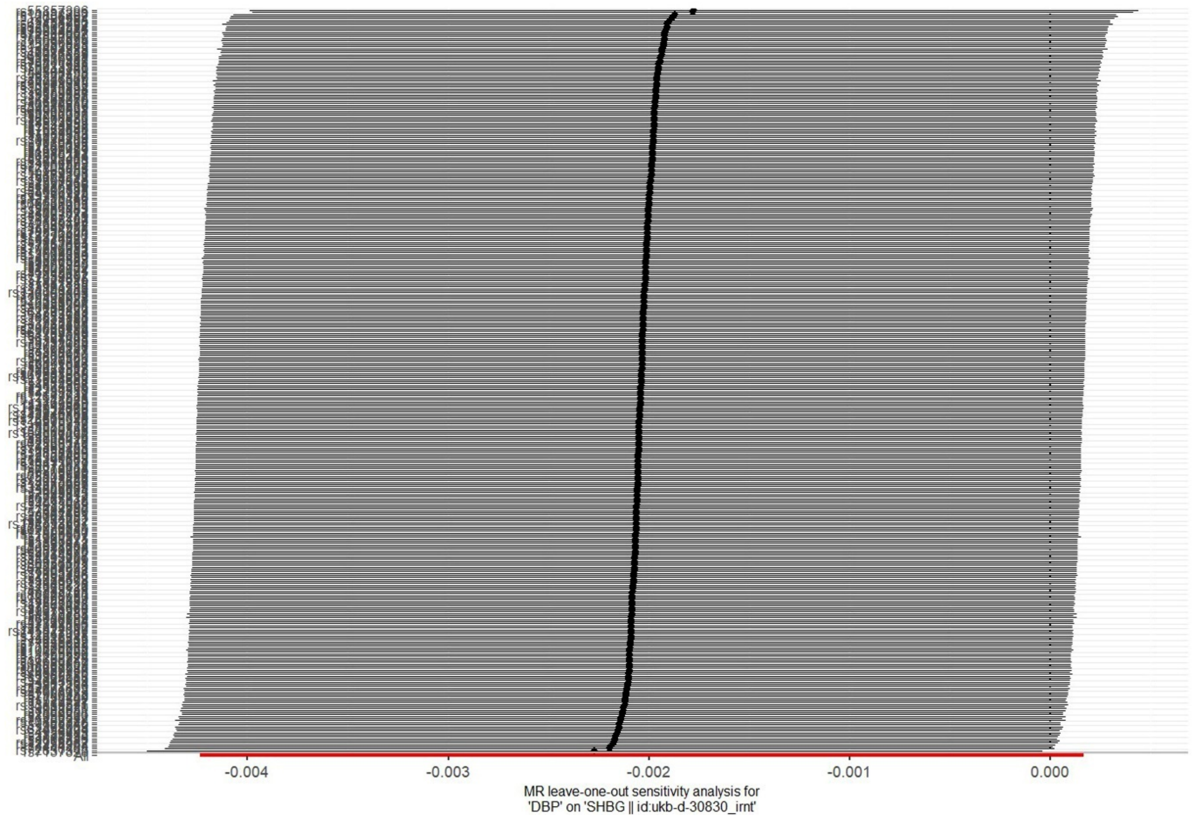

Supplementary Figure S15 Leave-one-out analysis for the causal estimates of diastolic blood pressure and sex hormone-binding globulin

Supplementary references

1. Shim H, Chasman DI, Smith JD, Mora S, Ridker PM, Nickerson DA, Krauss RM, Stephens M.. A multivariate genome-wide association analysis of 10 LDL subfractions, and their response to statin treatment, in 1868 Caucasians. PLoS One **2015**, 10(4): e0120758.
2. Pierce BL, Ahsan H, Vanderweele TJ. Power and instrument strength requirements for Mendelian randomization studies using multiple genetic variants. Int J Epidemiol **2011**, 40(3): 740-752.
3. Greco M FD, Minelli C, Sheehan NA, Thompson JR. Detecting pleiotropy in Mendelian randomisation studies with summary data and a continuous outcome. Stat Med **2015**, 34(21): 2926-2940.
4. Zhan Y, Karlsson IK, Karlsson R, Tillander A, Reynolds CA, Pedersen NL, Hägg S. Exploring the Causal Pathway From Telomere Length to Coronary Heart Disease: A Network Mendelian Randomization Study. Circ Res **2017**, 121(3): 214-219.
5. Dastani Z, Hivert MF, Timpson N, Perry JR, Yuan X, Scott RA, Henneman P, Heid IM, Kizer JR, Lyytikäinen LP, et al. Novel loci for adiponectin levels and their influence on type 2 diabetes and metabolic traits: a multi-ethnic meta-analysis of 45,891 individuals. PLoS Genet **2012**, 8(3): e1002607.
6. Higgins JP, Thompson SG. Quantifying heterogeneity in a meta-analysis. Stat Med **2002**, 21(11): 1539-1558.
7. Higgins JP, Thompson SG, Deeks JJ, Altman DG. Measuring inconsistency in meta-analyses. BMJ **2003**, 327(7414): 557-560.
8. Skrivankova VW, Richmond RC, Woolf BAR, Yarmolinsky J, Davies NM, Swanson SA, VanderWeele TJ, Higgins JPT, Timpson NJ, Dimou N, et al. Strengthening the Reporting of Observational Studies in Epidemiology using Mendelian Randomization (STROBE-MR) Statement. JAMA **2021**, 326(16): 1614-1621.
9. Skrivankova VW, Richmond RC, Woolf BAR, Davies NM, Swanson SA, VanderWeele TJ, Timpson NJ, Higgins JPT, Dimou N, Langenberg C, et al. Strengthening the Reporting of Observational Studies in Epidemiology using Mendelian Randomisation (STROBE-MR): Explanation and Elaboration. BMJ **2021**, 375: n2233.
